# A New Type of Na^+^-Driven ATP Synthase Membrane Rotor with a Two-Carboxylate Ion-Coupling Motif

**DOI:** 10.1371/journal.pbio.1001596

**Published:** 2013-06-25

**Authors:** Sarah Schulz, Marina Iglesias-Cans, Alexander Krah, Özkan Yildiz, Vanessa Leone, Doreen Matthies, Gregory M. Cook, José D. Faraldo-Gómez, Thomas Meier

**Affiliations:** 1 Department of Structural Biology, Max Planck Institute of Biophysics, Frankfurt am Main, Germany; 2 Department of Microbiology and Immunology, Otago School of Medical Sciences, University of Otago, Dunedin, New Zealand; 3 Theoretical Molecular Biophysics Group, Max Planck Institute of Biophysics, Frankfurt am Main, Germany; 4 Cluster of Excellence “Macromolecular Complexes,” Goethe University of Frankfurt, Frankfurt am Main, Germany; University of Zurich, Switzerland

## Abstract

The anaerobic bacterium *Fusobacterium nucleatum* uses glutamate decarboxylation to generate a transmembrane gradient of Na^+^. Here, we demonstrate that this ion-motive force is directly coupled to ATP synthesis, via an F_1_F_o_-ATP synthase with a novel Na^+^ recognition motif, shared by other human pathogens. Molecular modeling and free-energy simulations of the rotary element of the enzyme, the c-ring, indicate Na^+^ specificity in physiological settings. Consistently, activity measurements showed Na^+^ stimulation of the enzyme, either membrane-embedded or isolated, and ATP synthesis was sensitive to the Na^+^ ionophore monensin. Furthermore, Na^+^ has a protective effect against inhibitors targeting the ion-binding sites, both in the complete ATP synthase and the isolated c-ring. Definitive evidence of Na^+^ coupling is provided by two identical crystal structures of the c_11_ ring, solved by X-ray crystallography at 2.2 and 2.6 Å resolution, at pH 5.3 and 8.7, respectively. Na^+^ ions occupy all binding sites, each coordinated by four amino acids and a water molecule. Intriguingly, two carboxylates instead of one mediate ion binding. Simulations and experiments demonstrate that this motif implies that a proton is concurrently bound to all sites, although Na^+^ alone drives the rotary mechanism. The structure thus reveals a new mode of ion coupling in ATP synthases and provides a basis for drug-design efforts against this opportunistic pathogen.

## Abstract 2

Multi-disciplinary methods reveal a novel type of ion binding in the rotor ring of the F_1_F_o_-ATP synthase from the opportunistic pathogen *Fusobacterium nucleatum*.

## Author Summary

Essential cellular processes such as biosynthesis, transport, and motility are sustained by the energy released in the hydrolysis of ATP, the universal energy carrier in living cells. Most ATP in the cell is produced by a membrane-bound enzyme, the ATP synthase, through a rotary mechanism that is coupled to the translocation of ions across the membrane. The majority of ATP synthases are energized by transmembrane electrochemical gradients of protons (proton-motive force), but a number of organisms, including some important human pathogens, use gradients of sodium ions instead (sodium-motive force). The ion specificity of ATP synthases is determined by a membrane-embedded sub-complex, the c-ring, which is the smallest known biological rotor. The functional mechanism of the rotor ring and its variations among different organisms are of wide interest, because of this enzyme's impact on metabolism and disease, and because of its potential for nanotechnology applications. Here, we characterize a previously unrecognized type of Na^+^-driven ATP synthase from the opportunistic human pathogen *Fusobacterium nucleatum*, which is implicated in periodontal diseases. We analyzed this ATP synthase and its rotor ring through a multi-disciplinary approach, combining cell-growth and biochemical assays, X-ray crystallography and computer-simulation methods. Two crystal structures of the membrane rotor were solved, at low and high pH, revealing an atypical ion-recognition motif mediated by two carboxylate side-chains. This motif is shared by other human pathogens, such as *Mycobacterium tuberculosis* or *Streptococcus pneumonia*, whose ATP synthases are targets of novel antibiotic drugs. The implications of this ion-recognition mode on the mechanism of the ATP synthase and the cellular bioenergetics of *F. nucleatum* were thus examined. Our results provide the basis for future pharmacological efforts against this important pathogen.

## Introduction

Synthesis of ATP, the most prominent energy source in biological cells, is largely mediated by the ATP synthase, an enzyme that resides in the membranes of bacteria, mitochondria, and chloroplasts. This enzyme catalyzes the phosphorylation of ADP by a rotary mechanism powered by a transmembrane electrochemical gradient, or ion-motive force, of either H^+^ or Na^+^ (proton-motive force [PMF] or sodium-motive force [SMF], respectively). The ATP synthase consists of two sub-complexes: the water-soluble F_1_ sector [1],[2], which harbors the catalytic centers, and the membrane-embedded F_o_ complex, which mediates ion translocation across the membrane. These functionally distinct units are mechanically coupled by two additional elements, referred to as central and peripheral stalks [3],[4].

In the F_o_ sector, eight to 15 copies of subunit c are assembled into a closed ring [5], which rotates around its axis as ions permeate across the enzyme. The c-ring harbors a series of identical ion-binding sites, typically one per c-subunit, which selectively recognize the coupling ion [6]–[8]. Ion binding is facilitated by a conserved carboxylic amino acid, usually glutamate; however, it is the neighboring chemical groups in the protein side-chains and backbone, and sometimes a bound water molecule [9]–[11] that ultimately determine the specificity of the c-ring binding sites [8]. Na^+^ specific sites typically involve an intricate hydrogen-bonded network of polar groups, while H^+^-binding sites are simpler, and consist mainly of hydrophobic moieties. Either way, one complete rotation of the c-ring results in the translocation of one ion per binding site and the production of three ATP molecules [12],[13]; the stoichiometry of the c-ring thus defines the ion-to-ATP ratio of the enzyme, i.e., the minimum ion-motive force required for ATP synthesis [14].

In this study, we characterize the structure, ion specificity, and stoichiometry of the c-ring of the ATP synthase from *F. nucleatum*, a Gram-negative bacterium implicated in the etiology of periodontal diseases. *F. nucleatum* grows anaerobically, using amino acids as the preferred carbon source [15]. In particular, glutamate fermentation involves the glutaconyl-CoA decarboxylase, which uses the free energy of decarboxylation to generate a SMF across the cytoplasmic membrane [16],[17]. Comparison of the amino-acid sequence of the *F. nucleatum* c-subunit with those of other Na^+^-driven ATP synthases suggests that *F. nucleatum* utilizes the SMF directly to produce ATP (Figure S1), but this remains to be experimentally demonstrated. Sequence analysis also suggests that ion coordination in the *F. nucleatum* c-ring could involve not only one but possibly two carboxyl side-chains. This is an unusual and interesting feature, shared by other pathogenic bacteria, whose mechanistic implications are unclear. It is conceivable that the second carboxyl group could alter the assumed ion specificity of the c-ring, the ion-to-ATP ratio, or that it confers a novel coupling or regulatory mechanism to the enzyme [18]. It is also unknown what c-subunit stoichiometry is characteristic of *F. nucleatum*. Crystal structures have revealed a wide range of possible stoichiometries in H^+^-coupled c-rings (reviewed in [5]); whether Na^+^-dependent c-rings from F-type ATP synthases are similarly diverse is unclear, since to date only one such ring has been characterized structurally at high resolution [6]. To clarify these questions, we have isolated the *F. nucleatum* F_1_F_o_-ATP synthase, and analyzed its ion-coupling mechanism using microbiological, biochemical, structural and computer-simulation methods, both classical and quantum-mechanical.

## Results

### Predicted Structure of the Ion-Binding Site in the *F. nucleatum* c-ring

To gain insights into the ion specificity of the *F. nucleatum* ATP synthase, a structural model of its c-ring was created by homology modeling and molecular dynamics simulations (Figure S2). The structure of the c-ring from *Ilyobacter tartaricus* was used as template [6],[9]. The resulting model thus consists of 11 c-subunits, with an ion-binding site in between each pair of adjacent subunits. Each of these sites includes two glutamate residues, namely Glu32 and Glu65. Glu65, in the C-terminal helix, is the conserved carboxylate found in other c-subunits. Glu32, in the N-terminal helix, is the additional carboxylate found only in selected species.

**Figure 1:**
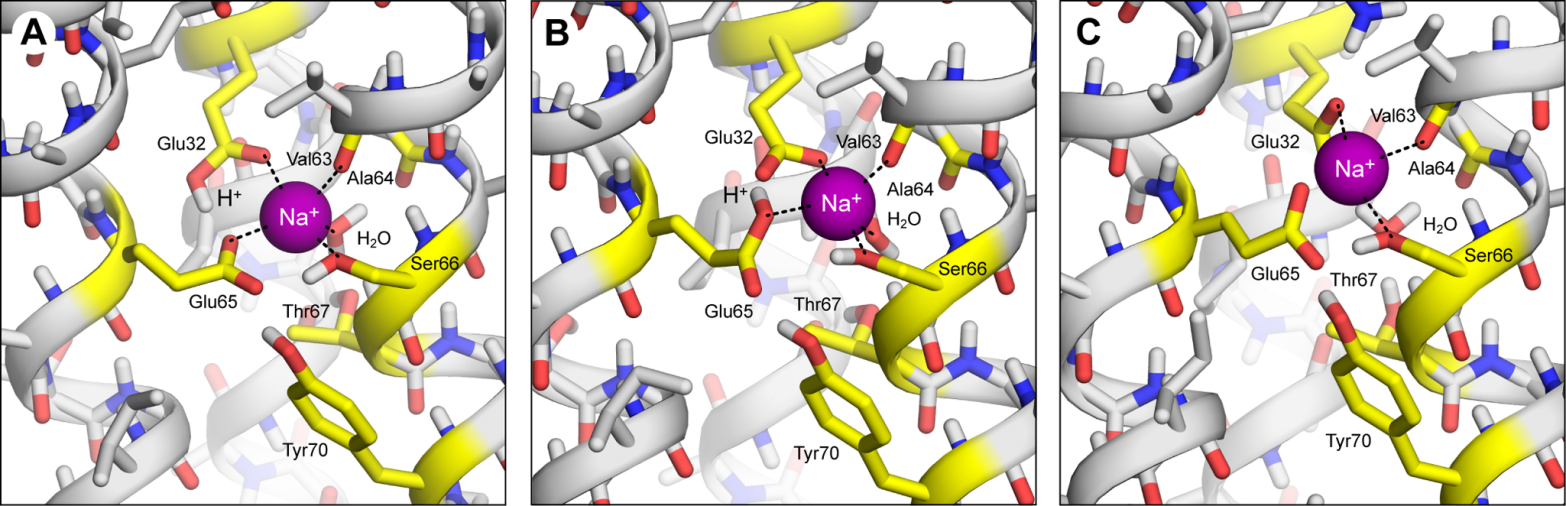
Alternative models of the ion-binding sites in the *F. nucleatum* c-ring, with Na^+^ bound. The ion-coordinating groups in the protein are indicated with dashed lines. Hydrogen atoms in non-polar groups are omitted for clarity. (A) Model with Glu32 protonated in *cis* and Glu65 in the deprotonated state (model A), predicted to be the most likely (see Results). (B) Model with Glu32 deprotonated and Glu65 protonated (model B). In this model, the proton bound to Glu65 was initially modeled in *cis*, as this is the most probable geometry for the isolated side-chain. However, within 250 ps of the start of the simulation, the carboxyl group of Glu65 switched to *trans*. (C) Model with Glu32 and Glu65 in the deprotonated state (model C). During the simulation of this model, the electrostatic repulsion between Glu32 and Glu65 caused the latter to displace outwards, away from the Na^+^ ion, which becomes coordinated by Glu32 in a bi-dentate configuration. Note the bound water reorients and no longer contributes to Na^+^ coordination.

The c-ring was initially modeled in the Na^+^-bound state. To identify the most plausible conformation of the ion-coordination sphere we constructed several models in alternate protonation states of Glu32 and Glu65, and assessed their likelihood using all-atom simulations of the complete c-ring in a lipid membrane, as well as quantum-mechanical energy calculations for a reduced model of the binding site. In the first of these models (model A), Glu32 is protonated (in *cis*) and Glu65 is deprotonated (Figure 1A); in this configuration, the binding site is highly similar to that in the crystal structure of the *I. tartaricus* c-ring [6],[9], except that in that structure Glu32 is replaced by glutamine. Na^+^ is coordinated directly by oxygen atoms in the side-chains of Glu65, Glu32, and Ser66, as well as by the backbone carbonyl of Val63 and a bound water molecule, which also interacts with Thr67. The proton bound to Glu32 mediates a hydrogen bond to Glu65, which is the acceptor of two more hydrogen bonds, donated by Tyr70 and Ser66. In a second model (model B), Glu32 is deprotonated while Glu65 is protonated (initially in *cis*). In simulation, this model evolves in time towards a conformation similar to that in model A, with the bound proton (which switches to *trans*) donating a hydrogen bond to Glu32, and Na^+^ penta-coordinated (Figure 1B). Lastly, in a third model (model C), both Glu32 and Glu65 were set in the deprotonated state (Figure 1C). This configuration leads to a noticeably different conformation of the binding site, in which only Glu32 and Ser66 coordinate the Na^+^ ion, while Glu65 is displaced outwards and the bound water molecule reorients.

**Figure 2:**
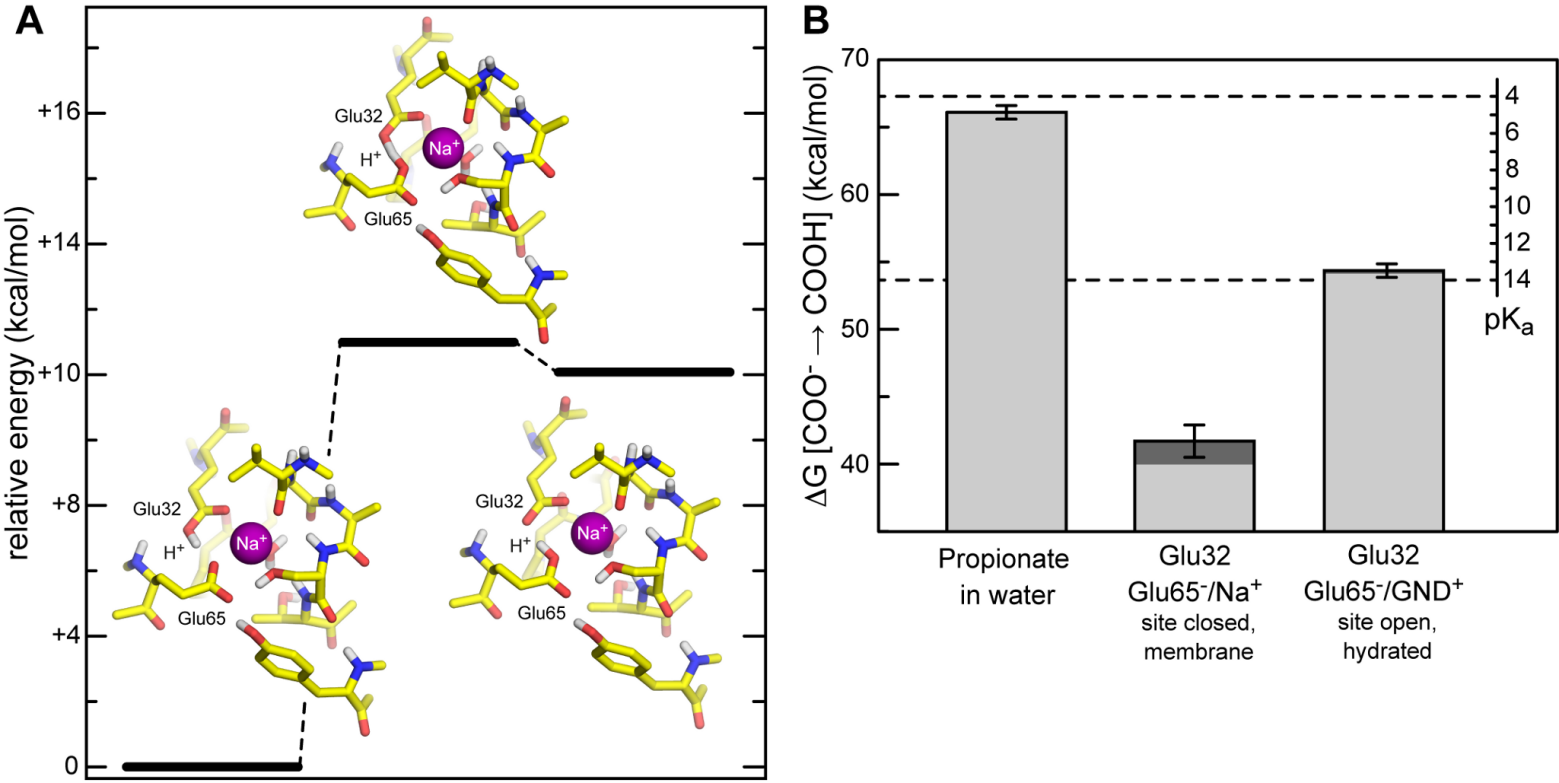
Likelihood of protonation of the Na^+^-loaded binding sites in the *F. nucleatum* c-ring, and most probable configuration. (A) Energetics of proton transfer between Glu65 and Glu32 (models A and B in Figure 1), with a Na^+^ concurrently bound, based on Hartree-Fock quantum-mechanical calculations for a reduced model of the binding site. The potential energy values plotted correspond to the optimized geometries in each case. The geometry and potential energy of the transition state are also provided. (B) Calculated free-energy gain associated with the protonation of Glu32, relative to a side-chain analog in solution (propionate); the formation energy of the chemical bond is omitted, as this is expected to be constant. Calculated values are translated into a pK_a_ scale, by setting the free-energy value for propionate to its known pK_a_ value, i.e., 4.9. The protonation of Glu32 is studied either in the Na^+^-bound, closed conformation of the c-ring binding sites (Figure 1A); or in an open, hydrated conformation (Figure 9). In both cases, to the values calculated by FEP/MD (light grey) we added a correction calculated with Poisson electrostatic theory on account of the membrane polarizability (dark grey).

To determine whether model A is more or less probable than model B, we assessed the energetics of proton transfer between Glu32 and Glu65 using Hartree-Fock quantum-mechanical calculations (Figure 2A). These calculations demonstrate that model A is more energetically favorable than model B, by about 10 kcal/mol. That is, if a proton were to be bound to the site, it would be localized on Glu32. To determine whether model A is more or less probable than model C, i.e., whether a proton indeed resides in the binding site along with Na^+^, we carried out a calculation of the pK_a_ of Glu32, in the context of the complete c-ring with bound Na^+^, using Free-Energy Perturbation (FEP) simulations. This calculation shows that the pK_a_ of Glu32 is markedly shifted upwards, relative to its intrinsic value in solution (Figure 2B). Thus, we conclude that in the membrane-embedded, Na^+^-loaded binding sites of the *F. nucleatum* c-ring, Glu32 is protonated under physiological conditions. Our predicted structure of the Na^+^-bound state is therefore that in model A; i.e., both a sodium ion and a proton are concurrently bound to the same binding site, with the H^+^ bound to Glu32.

### Predicted Ion Selectivity of the *F. nucleatum* c-ring

**Figure 3:**
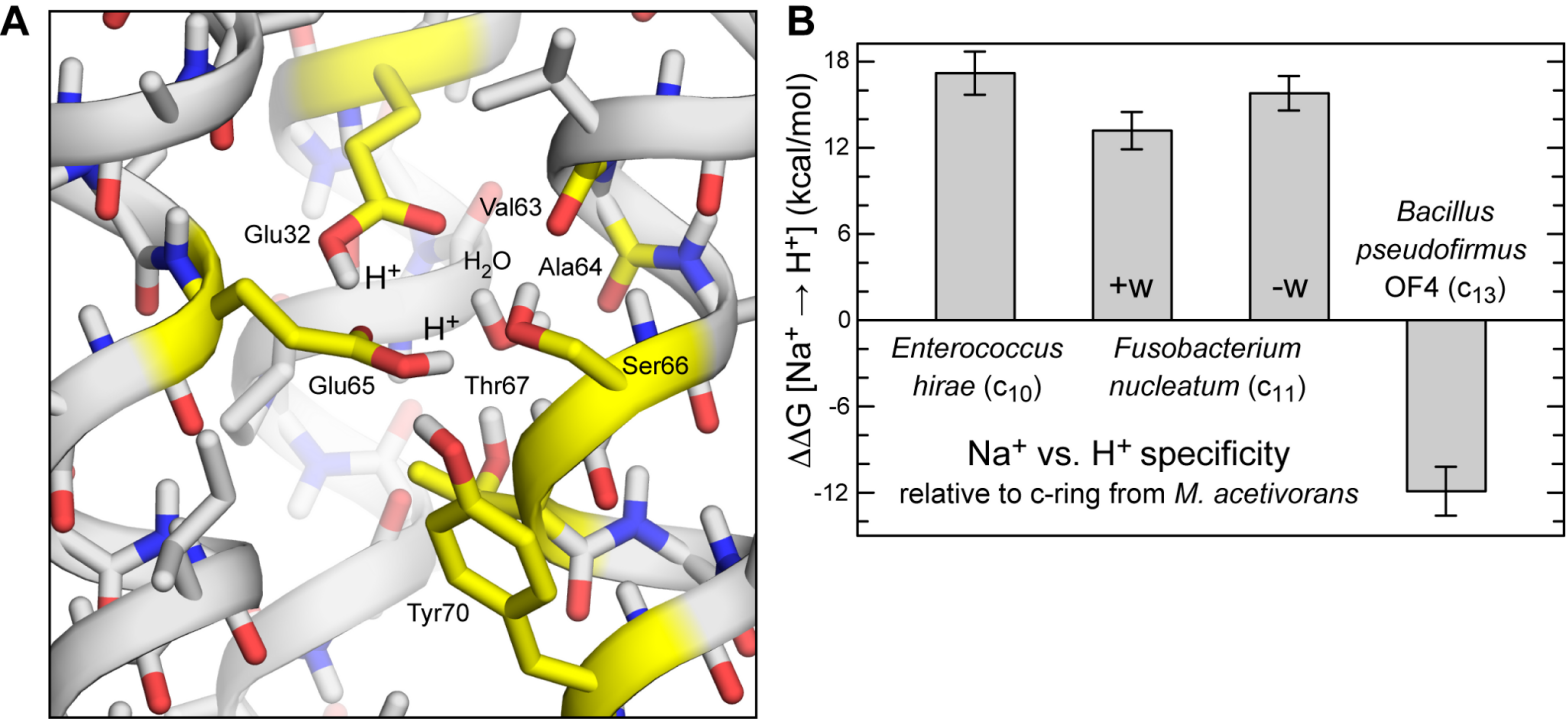
Calculated ion selectivity of the *F. nucleatum* c-ring. (A) Predicted structure of the ion binding sites in the absence of Na^+^, with two H^+^ bound. Hydrogen atoms in non-polar groups are omitted for clarity. Note the binding site harbors two H^+^, one bound to Glu32 and another to Glu65 (both in *cis*). Two alternative configurations, one of which lacks a bound water molecule, are also shown in Figure S3. (B) Calculated free-energy of selectivity for H^+^ vs. Na^+^ of the *F. nucleatum* c-ring binding sites, relative to other c-rings previously characterized. The Na^+^ state is that rendered in Figure 1A; note the bound water molecule. Two possible H^+^-bound states are considered, in which the water molecule is either preserved (+w), as in Figure 3A, or removed (−w), as in Figure S3B.

The ion selectivity of c-rings in which only one ion can occupy the binding sites is defined by the difference in the binding free-energy of either H^+^ or Na^+^ [8],[19],[20]. Because in the *F. nucleatum* c-ring one additional H^+^ resides in the binding site in the Na^+^ state, the analogous free-energy difference is that between a state with two H^+^ bound and another with one Na^+^ and one H^+^. Thus, to examine the question of whether the *F. nucleatum* ATP synthase is driven by a SMF or a PMF, we re-modeled and simulated the structure of the c-ring with two H^+^ bound to Glu65 and Glu32, respectively (Figures 3A and S2), and used additional free-energy calculations to assess the likelihood of this doubly protonated state, relative to a state with one Na^+^ and one H^+^ bound to the site.

Figure 3B shows the computed free energy of selectivity of the *F. nucleatum* rotor in reference to other c-rings characterized previously [11],[19],[20]. Two alternative models of the H^+^-bound state were considered (Figures 3A and S2), differing on whether the structural water molecule present in the Na^+^-bound state is included in the site or not. In both cases, the calculations show that the specificity of the *F. nucleatum* c-ring is in stark contrast to that of H^+^-driven ATP synthases. For example, relative to the c-ring of *B. pseudofirmus* OF4, the selectivity for H^+^ of the *F. nucleatum* c-ring is drastically diminished in favor of Na^+^, by about 26 kcal/mol, i.e., more than ten orders of magnitude (Figure 3B). Instead, the computations indicate that the ion specificity of the *F. nucleatum* rotor is comparable to that of the V-type ATPase from *Enterococcus hirae*, which has been experimentally shown to be very weakly H^+^ selective [21]. In particular, the *F. nucleatum* c-ring is more selective for H^+^ and opposed to Na^+^ than that of *E. hirae*, by ∼1–3 kcal/mol, i.e., up to ∼100-fold. Nevertheless, because in the physiological environment of *F. nucleatum* (i.e., the human mouth flora) Na^+^ is in excess over protons at least by five orders of magnitude, our prediction is therefore that one Na^+^ and one H^+^, rather than two H^+^, will typically occupy the ion-binding sites of the *F. nucleatum* c-ring. For the same reason the *E. hirae* ATPase functions as a Na^+^ pump under physiological conditions. Thus, we conclude that in *F. nucleatum* the SMF is used to drive ATP synthesis directly.

### Effect of Ionophores and Related Inhibitors on *F. nucleatum* Growth

To begin to assess the specificity of *F. nucleatum* ATP synthase experimentally, we first tested the effect of various protonophores, ionophores, and ATP synthase inhibitors on anaerobic growth (Figure S4). Growth was inhibited by the protonophores carbonyl cyanide m-chlorophenylhydrazone (CCCP), 2,4-dinitrophenol (DNP), and 3,3′,4′,5-tetrachlorosalicylanilide (TCS), but the sensitivity to these agents varied, with TCS being the most potent. Monensin, a monovalent cation ionophore, was also a potent growth inhibitor of *F. nucleatum*. Amiloride and its more hydrophobic derivative 5-(*N*-ethyl-*N*-isopropyl) amiloride (EIPA), are blockers of Na^+^ channels and Na^+^/H^+^ antiporters, and both compounds inhibited the growth of *F. nucleatum*. The membrane-permeable ATP synthase inhibitor *N*,*N*′-dicyclohexylcarbodiimide (DCCD) also inhibited growth, but tributyltin chloride (TBT-Cl) had no significant effect (Figure S5). Taken together, these data demonstrate that *F. nucleatum* cells require both a PMF and a SMF to grow, and that classical ATP synthase inhibitors slow down growth. However, these data do not clarify whether the *F. nucleatum* ATP synthase utilizes the PMF or the SMF to sustain ATP production.

### Characterization of the *F. nucleatum* ATP Synthase

To clarify what energy source is utilized by the *F. nucleatum* ATP synthase, we set out to specifically characterize the enzyme, both in inverted membrane vesicles from *F. nucleatum* and purified. The ATP hydrolysis activity of the inverted membranes was in the range 0.09–0.15 units/mg protein (1 unit = 1 µmol ATP hydrolyzed/min), and exhibited a weak pH dependence, with a pH optimum of 7.5–8.5 (Figure S6A). This ATPase activity was strongly inhibited by DCCD (unpublished data), which covalently modifies the carboxyl side-chain of the c-ring ion-binding sites (here Glu65). ATP synthesis in inverted membrane vesicles could be driven by a SMF and under these conditions ATP synthesis was sensitive to both the sodium ionophore monensin and DCCD (Figure S6B). No significant ATP synthesis could be detected in the absence of Na^+^, even in the presence of a valinomycin-induced K^+^ diffusion potential. However, a chemical gradient of Na^+^ was not sufficient to drive ATP synthesis in the absence of a membrane potential. These data support the presence of a Na^+^-coupled F-type ATP synthase in membrane vesicles of *F. nucleatum*.

We next purified the ATP synthase, ultimately achieving a 38-fold purification (Table S1). Silver-stained SDS-gels and MALDI were used to confirm that all constituent subunits were present in the purified sample (Figure S7). The ATPase activity of the purified enzyme was stable over several days (Figure S8A). The apparent K_m_ for Mg^2+^ and ATP were 0.64 mM and 0.25 mM, respectively (Figure S8B–S8C). CaCl_2_ could not replace MgCl_2_, since no activity was detected at increasing concentrations of CaCl_2_ (unpublished data).

**Figure 4:**
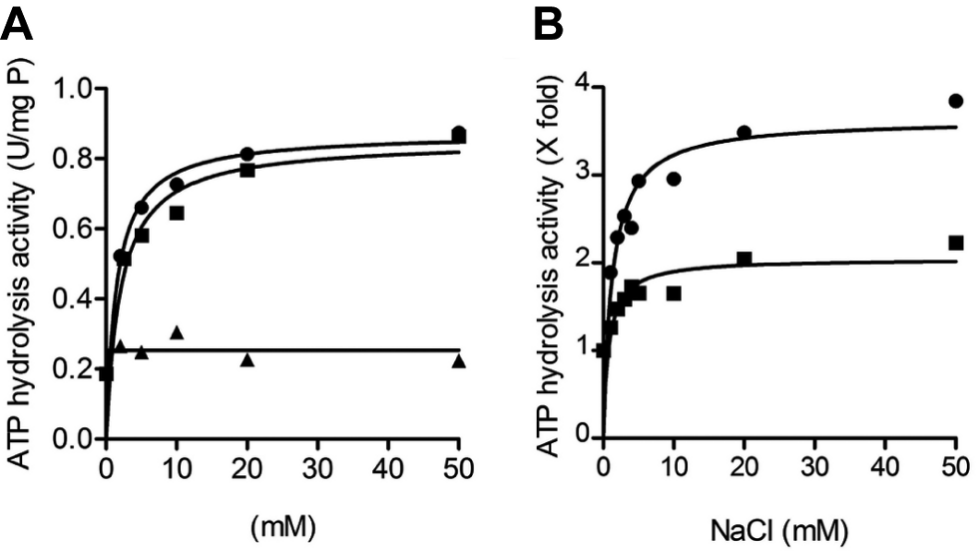
Catalytic activity of the purified F_1_F_o_-ATP synthase from *F. nucleatum*. (A) Activation of the *F. nucleatum* ATP synthase by Na^+^, Li^+^, and K^+^ ions (Na^+^, •; Li^+^, ▪; and K^+^, ▴). (B) Activation of the ATP synthase by Na^+^ ions at pH 7.5 (•) and pH 9.0 (▪). The ATP hydrolysis activity was determined using the ATP-regenerating assay (120–140 µg protein), at 37°C. Activity in units/mg of protein (1 unit = 1 µmol ATP hydrolysed/min). 100% of activity corresponds 1–2 units/mg at pH 7.5 and 0.6–0.72 units/mg at pH 9.0. The assay mixture contained 50 mM MOPS, 2 mM MgCl_2_ (pH 7.5) in (A) and 50 mM MES-MOPS-Tris, 2 mM MgCl_2_ in (B). The values plotted are representative of at least two biological replicates; the statistical variance was less than 20%.

**Figure 5:**
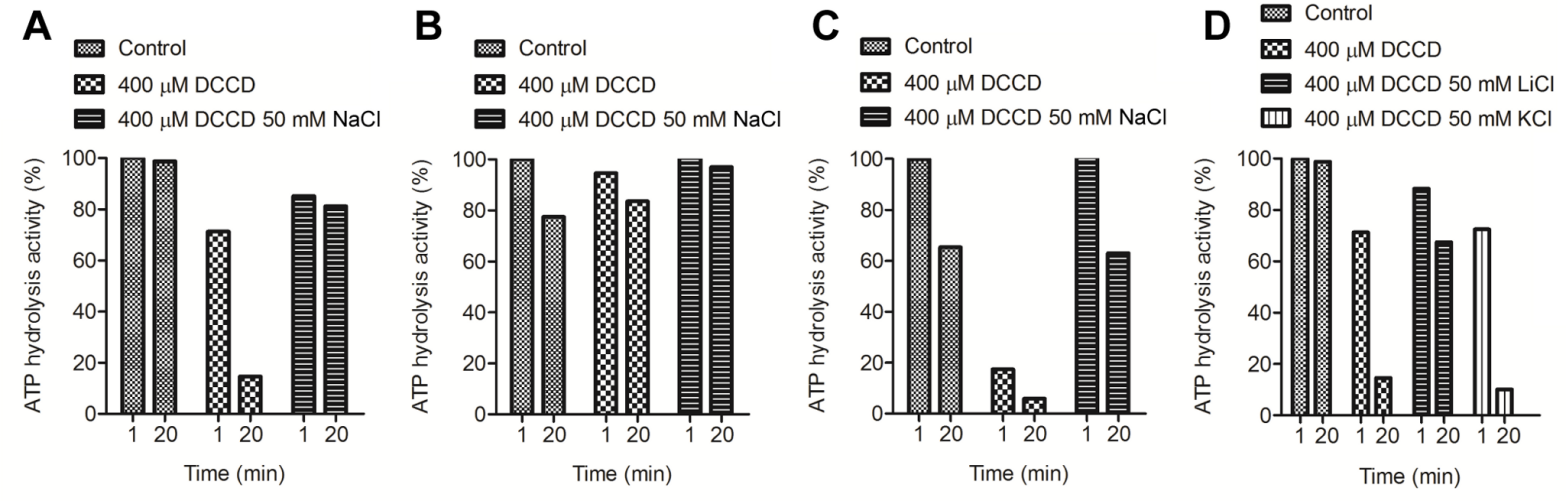
Protective effect of Na^+^ and Li^+^ against DCCD inhibition of the purified *F. nucleatum* ATP synthase. The purified protein (120–140 µg) was incubated at 25°C for 20 min in the presence of either 400 µM DCCD or 400 µM DCCD and 50 mM NaCl in 50 mM MES-MOPS-Tris, 2 mM MgCl_2_, (A) at pH 7.5, (B) at pH 9, and (C) at pH 6.5. (D) NaCl was substituted by either LiCl or KCl in 50 mM MES-MOPS-Tris, 2 mM MgCl_2_, pH 7.5. 100% enzyme activity corresponds to 1–2 units/mg of protein. The values plotted are representative of at least two to three individual experiments; the statistical variance was less than 20%.

Two cornerstone properties of all Na^+^-coupled ATP synthases are the ability to be specifically stimulated by low amounts of Na^+^ (or Li^+^), and the protective effect of Na^+^ against DCCD inhibition [22],[23]. Both these properties were apparent in the *F. nucleatum* ATP synthase (Figures 4 and 5). While K^+^ had no stimulatory effect on the ATPase activity, Na^+^ (and Li^+^) stimulated maximally at low millimolar concentrations (Figure 4A), both at neutral and high pH (Figure 4B). The ATPase activity of the enzyme was inhibited by DCCD in a concentration-dependent manner (Figure S9). However, even with high concentrations of DCCD (400 µM), 50 mM Na^+^ (or 50 mM Li^+^, but not K^+^) strongly protected the enzyme from inhibition (Figure 5). The protective effect of Na^+^ was most apparent at lower pH values, consistent with the fact that DCCD modification requires protonation of Glu65. These observations are strongly indicative of a specific binding site for Na^+^ in the c-ring of the *F. nucleatum* ATP synthase.

### Evidence of Na^+^ Binding to the Isolated *F. nucleatum* c-ring

We next purified the *F. nucleatum* c-ring using a novel heterologous expression system in *Escherichia coli* DK8 cells, which synthesizes a hybrid F_1_F_o_-ATP synthase with subunits F_1_-ab_2_ from *I. tartaricus* and the c-ring from *F. nucleatum*. From this hybrid F_1_F_o_ complex we isolated the c-ring, which migrates at ∼51 kDa on an SDS-polyacrylamide gel, disintegrating into c-monomers only upon tricholoroacetic acid (TCA) treatment (Figure S10). The expected mass of *F. nucleatum* c-subunit was confirmed by mass spectrometry (Figure S11).

**Figure 6:**
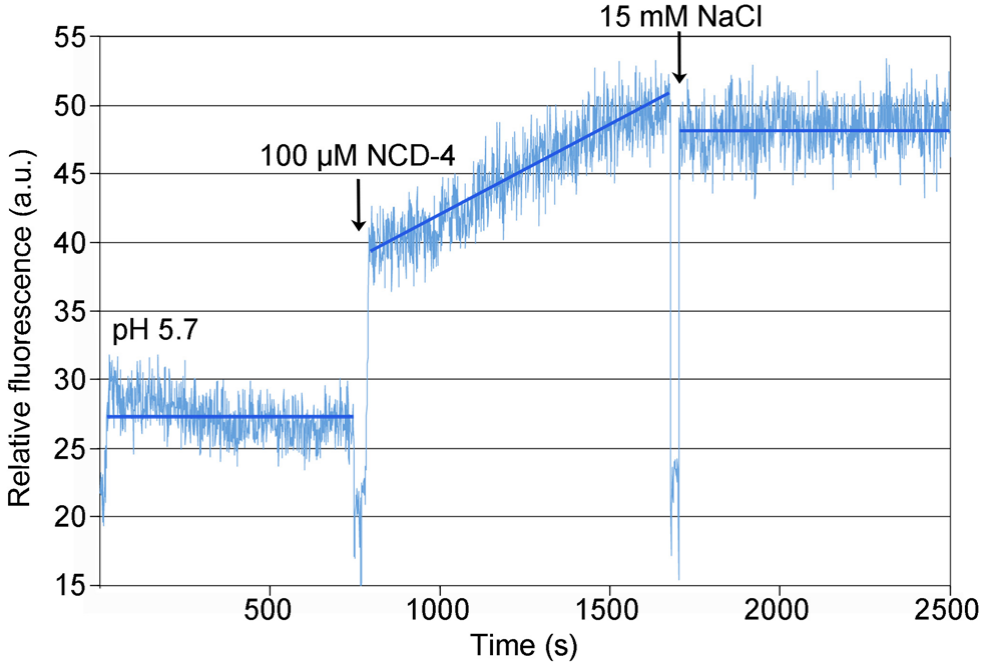
Kinetics of NCD-4 modification of detergent-solubilized c-rings from *F. nucleatum*. 100 µM NCD-4 was added to a sample of purified *F. nucleatum* c_11_ ring in MES buffer (pH 5.7) and 1.5% (w/v) n-octyl-β-D-glycoside. A continuous increase in fluorescence was measured upon reaction of NCD-4 with Glu65. Addition of 15 mM NaCl precluded further increase in the fluorescence. An extended control measurement, with no NaCl added, is shown in Figure S12.

To address the question of ion specificity, we exposed detergent-solubilized c-rings to *N*-cyclohexyl-*N*′-(4-(dimethylamino)-α-naphthyl)-carbodiimide (NCD-4), a fluorescent DCCD analogue, in the absence and presence of Na^+^ at pH 5.7. The reaction of NCD-4 with the c-ring resulted in a continuous increase of fluorescence (Figure 6). The reaction stopped immediately upon addition of only 15 mM NaCl, but continued linearly in a separate long-term control experiment in the absence of NaCl (Figure S12). These data indicate that NCD-4 modifies Glu65 in the isolated c-ring, and that Na^+^ ions inhibit this reaction, in clear contrast to similar measurements for H^+^-coupled c-rings, in which Na^+^ had no effect [8],[10]. The rapid protective effect of Na^+^ demonstrates that ions can access the binding sites of detergent-solubilized c-rings in less than a few seconds (pipetting time), consistent with a previous report for an analog fluorescent inhibitor [24]. It is also worth mentioning that the migration of intact and monomerized (TCA-treated) c-ring samples on an SDS-polyacrylamide gel, exposed to UV light, was significantly reduced after NCD-4 modification (Figure S13), indicating that NCD-4 becomes covalently bound to the c-ring.

### High-Resolution Structure of the *F. nucleatum* c_11_ Ring

**Figure 7:**
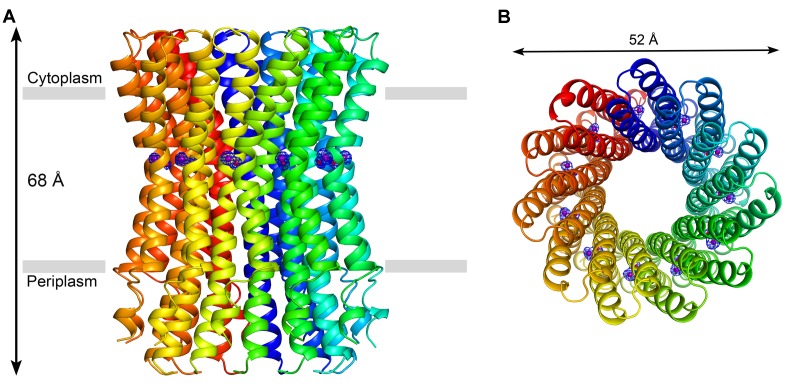
Crystal structure of the c_11_ ring of the *F. nucleatum* ATP synthase at 2.2 Å resolution. The pH of crystallization buffer was 5.3, and included 100 mM Na^+^. (A) Side view of the c-ring along the membrane plane. The c-subunits are displayed in different colors in ribbon representation. The transmembrane region, 35 Å in width, is indicated with grey bars. (B) View of the c-ring from the cytoplasm, along the perpendicular to the membrane. The sodium ion and the water molecule bound to each of the 11 ion-binding sites (purple and red spheres, respectively), are shown by F_obs_-F_calc_ omit electron-density maps at 3.2σ (blue meshes).

To obtain structural evidence of a Na^+^-binding site in the *F. nucleatum* ATP synthase, we solved the atomic structure of its c-ring by X-ray crystallography. Crystals of the c_11_ ring were first grown at pH 5.3 and one of the crystals yielded a complete 2.2 Å dataset (Table S2). In this crystal, one asymmetric unit contains two complete c-rings. Like the c-ring from *I. tartaricus* [6], the *F. nucleatum* rotor is made by 11 identical c-subunits (Figure 7). Each c-subunit consists of two transmembrane α-helices connected by a short loop, which would face the cytoplasmic side of the membrane; both N- and C-termini would therefore be in the periplasmic space. In the crystal, lattice contacts are established between the hydrophilic C-termini and the short loop.

**Figure 8:**
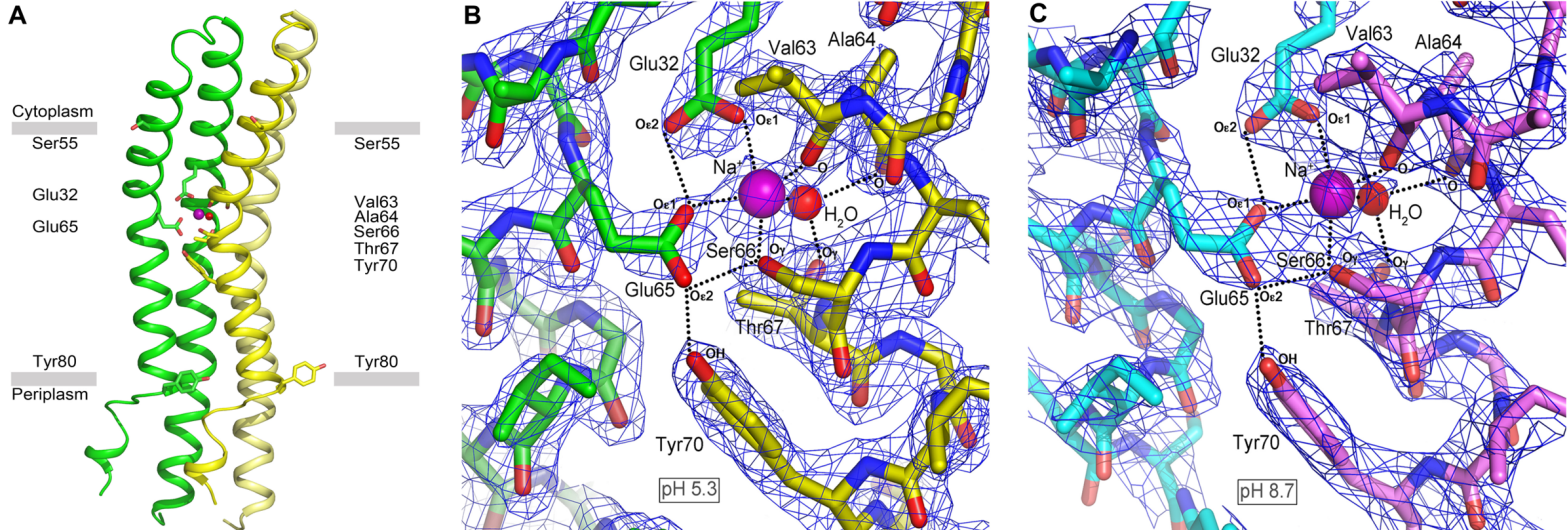
The Na^+^-binding site of the *F. nucleatum* c_11_ ring at pH 5.3 and 8.7. (A) A functional Na^+^-binding site consists of two adjacent c-subunits (in yellow and green). Residues discussed in the text are indicated. (B) Close-up of the Na^+^-binding site viewed from the membrane plane, from a crystal grown at pH 5.3 and 100 mM NaOAc; the resolution of the data is 2.2 Å. The Na^+^ and a structural water molecule are shown in purple and red spheres, respectively. The H-bond network and ion-protein interactions are indicated by dashed lines. Residues involved in Na^+^ binding are labeled. (C) Close-up of the Na^+^ binding site from a crystal grown at pH 8.7 and 100 mM buffer, viewed as in (B); the resolution is 2.6 Å. Electron density maps (2F_obs_-F_calc_, blue mesh) are shown at 1.9σ. In both cases Na^+^ ions can be discerned in the ion-binding sites, and consistently the key Glu65 side-chain is observed in the ion-locked conformation, while Glu32 is protonated.

The c_11_ ring is ∼68 Å in height and its outermost diameter, towards the cytoplasmic side, is ∼52 Å (Figure 7). The diameter of the ring is smallest at its middle (∼40 Å), i.e., at the level of the ion-binding sites, and therefore its shape resembles an hourglass. The transmembrane region of the protein appears to extend from Tyr80 to Ser55 on the periplasmic and cytoplasmic sides, respectively (Figure 8A). Within these limits, the c-ring surface is highly hydrophobic, while polar and charged amino acids are predominantly found on the surface facing the cytoplasm, as well as at the N- and C-termini. Like other c-rings, the *F. nucleatum* c_11_ ring features an inner pore (Figure 7B), mostly lined by hydrophobic residues. Detergent molecules can also be discerned on the periplasmic entrance of the pore, suggesting that this pore would be blocked by phospholipids (Figure S2B) in vivo. According to the mass spectrometry analysis (Figure S11), a small fraction of the c-subunits in our preparation is formylated at the N-termini, but apparently not in sufficient amounts to yield enough electron density from the diffraction data.

The c-ring was crystallized in the presence of near-physiological concentrations of Na^+^ (100 mM). To confirm the location of the Na^+^-binding sites, we calculated an omit electron-density map (Figure 7). This map reveals 11 binding sites, each located at one of the interfaces between c-subunits. A close-up view of these sites reveals an interaction network of several amino acids around the central density caused by Na^+^ (Figure 8B; Table 1). The arrangement of the Na^+^-binding site is highly similar to that in the *I. tartaricus* c_11_ ring [6],[9]. The most notable difference is the presence of a second glutamate side-chain, Glu32, in addition to the conserved Glu on the C-terminal helix (Glu65). Both side-chains contribute to Na^+^ coordination, via one oxygen atom of each carboxyl group. The other two oxygen atoms are at hydrogen-bonding distance of ∼2.7 Å from each other (Table 1). This arrangement suggests that a proton is bound to the site in addition to Na^+^, consistent with the free-energy calculations described above (Figure 2). In addition to Glu32 and Glu65, Ser66:Oγ and Val63:O from the adjacent c-subunit also contribute to Na^+^ coordination. Ser66:Oγ and Tyr70:OH also interact with Glu65, as hydrogen-bond donors, stabilizing the so-called ion-locked conformation of the site [6]. Finally, electron density for a water molecule is found within coordinating distance from the ion, with Thr67:Oγ and Ala64:O as potential hydrogen-bond acceptors.

**Table 1:** Inter-atomic distances at the Na^+^ binding sites of the *F. nucleatum* c-ring, in the crystals at pH 5.3 and 8.7, and in simulation.

From	To	Distance [Å]X-ray pH 5.3^a^	Distance [Å]X-ray pH 8.7^a^	Distance [Å]Simulation^b^
Na^+^	Glu32 Oε1	2.35±0.07	2.40±0.13	2.33±0.14
Na^+^	Val63 O	2.33±0.08	2.36±0.09	2.33±0.13
Na^+^	Glu65 Oε1	2.37±0.06	2.43±0.07	2.19±0.08
Na^+^	Ser66 Oγ	2.32±0.07	2.30±0.08	2.32±0.09
Na^+^	HOH	2.44±0.10	2.46±0.09	2.31±0.10
HOH	Ala64 O	2.61±0.12	2.65±0.17	2.72±0.12
HOH	Thr67 Oγ	2.69±0.15	2.77±0.19	2.82±0.13
Glu65 Oε1	Glu32 Oε2	2.66±0.06	2.67±0.09	2.60±0.08
Glu65 Oε2	Tyr70 OH	2.72±0.10	2.81±0.10	2.74±0.19
Glu65 Oε2	Ser66 Oγ	2.52±0.09	2.55±0.14	2.73±0.18

a Crystallographic values are averages, with the corresponding standard deviations, over the 11 binding sites in each of the two c-rings found in the asymmetric unit. The structural refinement was carried out without non-crystallographic symmetry restraints on the Na^+^ and HOH positions.

b The simulation values are time-averages extracted from the simulation of Model A (Figure 1A), over the 11 binding sites in the c-ring. The average structure of the c-ring backbone in simulation is superimposed on the crystal structure in Figure S14; the RMS difference is 0.74 Å.

We also investigated the influence of the environmental pH on the conformational state of the ion-binding site, by crystallizing the *F. nucleatum* c_11_ ring in NaOH-titrated buffer at pH 8.7. The resulting structure, at 2.6 Å resolution (Table 1), revealed again a locked conformation of the binding site identical to that at pH 5.3, with Na^+^ and H^+^ bound concurrently (Figure 8). As we observed in previous structural studies of c-rings [10],[25], the detergent used in these crystals provides a hydrophobic belt that effectively uncouples the binding sites from the pH or Na^+^ concentration conditions in the crystallization buffer, in a manner that likely resembles the shielding effect of a lipid membrane.

### Is the Additional H^+^ Bound to the c-ring Co-transported with Na^+^?

The finding that one Na^+^ and one H^+^ can concurrently occupy the binding sites in the *F. nucleatum* c_11_ ring raises the possibility that both ions are co-transported across the membrane as the ring rotates. This would imply that ATP synthesis in *F. nucleatum* is not only coupled to the SMF, but also to the PMF. More generally, the implication for other c-rings whose ion-binding sites include two carboxyl groups would be an ion-to-ATP ratio that is twice larger than what the c-subunit stoichiometry indicates (here 22/3 instead of 11/3).

In the operating enzyme, ion loading and release from the c-ring occurs when each of the c-subunits encounters the interface with the adjacent subunit-a, as the ring rotates within the membrane. No high-resolution structural data are available for subunit-a or its interface with the c-ring, but this interface is reportedly well hydrated [26],[27]. We have previously shown that hydration is sufficient to facilitate the reversible gating of the c-subunit binding sites, and to enable binding and unbinding of ions [25],[28]. In the open, ion-free state, the conserved glutamate projects away from the ring [25],[28],[29] and is believed to transiently engage a conserved arginine side-chain in the fourth transmembrane helix of subunit-a [30],[31].

**Figure 9:**
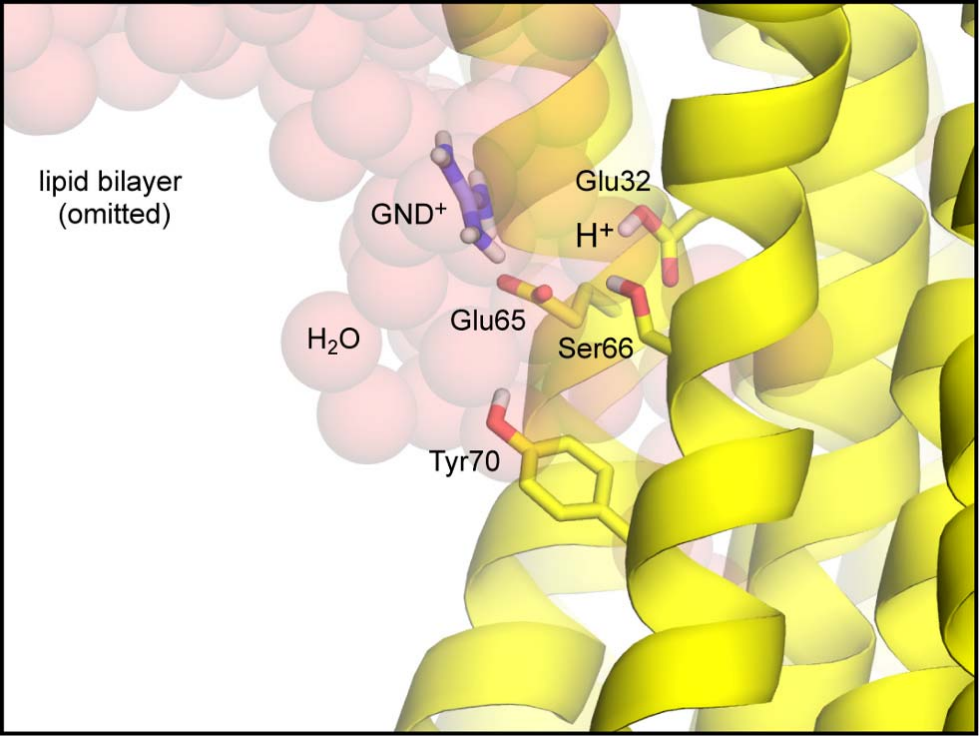
Simulation model of the open-state of the Na^+^-binding site in the *F. nucleatum* c-ring. The c-ring is shown in cartoon format (yellow), viewed from the membrane plane. Lipid molecules in the surrounding membrane bilayer are omitted for clarity. A crevice of water molecules (transparent red spheres) was modeled on the cytoplasmic side of the protein-lipid interface, so as to mimic the hydrated environment at the interface of the c-ring and subunit a, in the complete enzyme. A guanidinium ion (GND^+^) was added to the crevice, to mimic a conserved Arg side-chain in the fourth transmembrane helix of subunit a. Key side-chains in the open binding site, namely Glu65, Glu32, Ser66, and Tyr70, are shown as sticks (non-polar hydrogen atoms are omitted); these form an interaction network in the closed state (Figure 8), which in this open state is largely disrupted. Glu65 projects out of the binding site and interacts with the guanidinium ion; Glu32, however, is highly likely to remain protonated (Figure 2B).

From an electrostatic standpoint, the likelihood of concurrent release of Na^+^ and H^+^ from the *F. nucleatum* ring would be maximal in this hypothetical transient state, since negatively charged Glu65 would be more distant from Glu32 than in the closed state, while still being neutralized by the key arginine in subunit-a, instead of the bound Na^+^. To clarify whether the likelihood of this event is significant, we created an idealized structural model of this intermediate configuration (Figure 9). We then re-computed the pK_a_ of Glu32 using FEP simulations. As shown in Figure 2B, the upward shift in the calculated pK_a_ of Glu32 in this open, hydrated state is indeed significantly smaller than in the closed state, but nevertheless deprotonation remains very unlikely. This result implies that only Na^+^ would be released from the c-ring, and therefore translocated across the membrane, while the additional H^+^ are constitutively bound to the structure.

**Figure 10:**
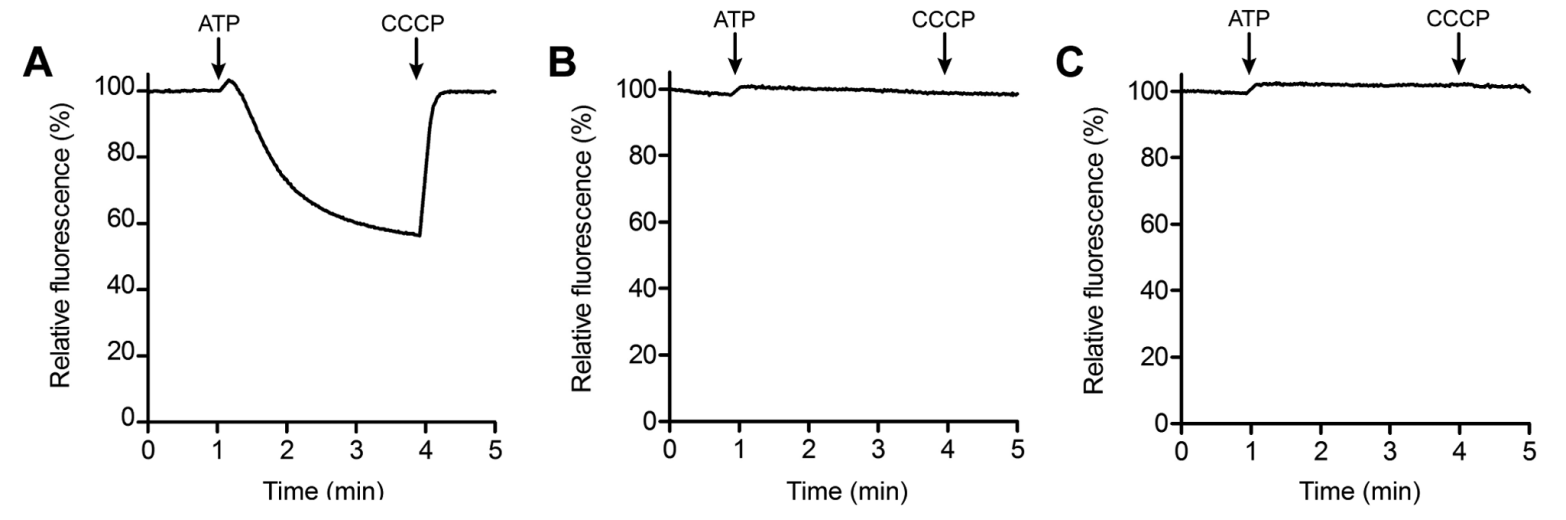
ATP-driven proton translocation in *E. coli* and *F. nucleatum* inverted membranes vesicles. Proton translocation was determined at 37°C by the quenching of acridine orange (AO) fluorescence. The reaction was initiated by the addition of 1.25 mM ATP and terminated with 30 µM CCCP at the times indicated by arrows. (A) *E. coli* native membranes in pH 7.5 buffer. (B) *F. nucleatum* native membranes in pH 6 buffer with 1.25 mM Na^+^-ATP; (C) Same as (B), with 10 mM NaCl added to the buffer.

To validate this conclusion experimentally, we examined the extent of proton uptake into inverted membrane vesicles from *F. nucleatum* and *E. coli* cells, driven by their respective ATP synthases, functioning in the ATP hydrolysis direction (see Methods). As expected, addition of ATP to the *E. coli* membranes resulted in the quenching of the fluorescence pH reporter acridine orange, due to H^+^ accumulation inside the vesicles (Figure 10A). This quenching was reversed by the addition of CCCP demonstrating that a pH gradient was indeed present. However, no discernible quenching was detected for *F. nucleatum*, under conditions in which its ATP synthase is active in hydrolysis. Specifically we tested buffers with Na^+^ concentrations of ∼2 mM and 10 mM, at pH values of 6 (Figure 10B and 10C) or lower (unpublished data). Under these conditions we expect ATP hydrolysis to be coupled to Na^+^ uptake into the vesicles, based on the above mentioned activity and inhibition measurements for both the purified enzyme (Figures 4 and 5) and the c-ring (Figure 6), and the fact that ATP synthesis in these membrane vesicles is Na^+^-dependent and sensitive to monensin (Figure S6B). However, proton co-transport was not detected in any case.

In sum, computations and measurements demonstrate that the protons that occupy the ion-binding sites in the *F. nucleatum* c-ring, concurrently with Na^+^, remain bound to the structure throughout the rotation of the ring, and therefore are not co-translocated with Na^+^ across the membrane.

## Discussion

### Mechanistic Implications of the Two-Carboxylate Ion-Binding Motif

We describe the structure of a novel type of Na^+^-binding site in the c-ring of the F_1_F_o_-ATP synthase from *F. nucleatum* and demonstrate that this enzyme is physiologically coupled to the SMF. The c-ring features two glutamate residues in each of its 11 ion-binding sites, instead of a single carboxylate side-chain as is typical. While such a motif has been observed in other Na^+^-binding proteins, and had been anticipated for c-rings in particular [9],[18], the implications in regard to the mechanism of ion transport and the specificity of the enzyme were unclear. A previous biochemical study of the PMF-driven ATP synthase from *E. coli* analyzed mutants with a two-carboxylate motif in the c-ring proton-binding sites, concluding that the engineered carboxylate (Asp24), which replaces a hydrophobic side-chain (Ala24), must be protonated for the enzyme to function, while it is the aspartate side-chain found in the wild-type enzyme (Asp61) that likely mediates H^+^ translocation [32]. The substitution of Ala24 by Asp or Glu, however, induces a pH dependence on the H^+^ transport activity in the physiological pH range, not observed for the wild-type enzyme. In a subsequent study of the two-carboxylate *E. coli* mutants, this dependence led to the proposal that the second carboxylate found in the binding sites of some c-rings might function as a regulatory high-pH sensor to inhibit the ion-translocation mechanism [18]. In light of our results, however, we interpret this pH dependence differently. In our view, it likely reflects a down-shift in the overall p*K*_a_ of the proton-binding sites in the mutagenized *E. coli* c-ring, as a result of the constitutive protonation of the added carboxylic side-chain, which naturally diminishes the affinity for a second proton (i.e., the one transported across the membrane), relative to the wild-type. This interpretation would also be consistent with the diminished rate of H^+^ translocation mediated by these two-carboxylate mutants. In support of our hypothesis that the second carboxylate group is constitutively protonated, we find that under physiological conditions the *F. nucleatum* ATP synthase mediates the translocation of Na^+^ only, i.e., protons are not co-transported during either ATP hydrolysis or ATP synthesis, even though they are concurrently bound to the c-ring binding sites, as the crystal structures and calculations demonstrate.

### Selectivity of c-ring Binding Sites with Two Carboxyl Side-Chains

The structural basis for the ion specificity of the ATP synthase can be rationalized on the basis of the amino acid composition of the c-ring binding sites, and the concentrations of the two competing ions in the environment [8]. This study indicates that the *F. nucleatum* c-ring preferentially uses Na^+^ as the coupling ion, but only under conditions in which Na^+^ is in large excess over H^+^, e.g., in physiological settings. The c-ring is in fact selective for H^+^, like all other rings characterized thus far. In particular it appears to be ∼100-fold more H^+^ selective than the ring of the Na^+-^driven V-type ATPase from *E. hirae*, which reportedly is almost non-selective [21]. The finding that the additional glutamate side-chain in the c-ring binding sites is constitutively protonated implies that its energetic contribution to ion selectivity is largely equivalent to that of a glutamine or asparagine side-chain at the same position. We expect this equivalence to hold true also for H^+^-driven ATP synthases with a double-carboxylate motif, such as those in *Streptococcus pneumoniae* [33] and *M. tuberculosis* (Figure S1). These c-rings probably bind two H^+^ concurrently to each site (as in Figure 3A), only one of which is likely to be transported. This perspective would also be consistent with a recent study of the ATP synthase from the archaeon *Methanosarcina acetivorans* [19], whose c-ring binding sites were also predicted to feature two glutamate side-chains, one of which was proposed to be constitutively protonated. The *M. acetivorans* ATP synthase is reportedly coupled to both Na^+^ and H^+^ translocation under physiological conditions [19]. However, this is not due to the deprotonation of the atypical carboxyl side-chain in the c-ring (the equivalent to Glu32 in *F. nucleatum*), but rather to the increased H^+^ specificity of its binding sites, relative to Na^+^-driven c-ring rotors (Figure 3B). Physiologically, i.e., with Na^+^ in large excess over H^+^, the more pronounced H^+^ selectivity of the *M. acetivorans* c-ring makes the likelihood of a state with two bound H^+^ comparable to that with one Na^+^ and one H^+^, in contrast to *F. nucleatum*, for which the latter is the most probable.

### The Undecameric Stoichiometry of Na^+^-Binding c-rings

Intriguingly, all Na^+^-coupled c-rings from F-type ATP synthases characterized so far consist of 11 α-helical hairpins, including those of *I. tartaricus*, *P. modestum*, *A. woodii*, *C. paradoxum* [24],[34]–[36], and now *F. nucleatum*. The only apparent commonality of these bacteria is that they grow under anaerobic conditions and in environments where Na^+^ is abundant (e.g., marine sediment). All these organisms thus use the SMF to sustain ATP production, but a phylogenetic relationship among them is not apparent. A more evident similarity, besides the near-identical amino acid composition of the Na^+^-binding sites, is a glycine-rich motif in the N-terminal helix of the c-subunit (Figure S1), which has been shown to have a strong influence on the stoichiometry of the c-ring [14],[37],[38]. It would be therefore tempting to hypothesize that the 11-hairpin architecture might reflect a universal feature of all Na^+^-driven c-rings in F-type ATP synthases. Nevertheless, studies of A- and V-type rotary ATPases indicate that Na^+^ specificity is compatible with other c-ring stoichiometries [20],[39],[40]. Consistently, single-point mutations of the glycine-motif in the c_11_ ring from *I. tartaricus* result in increased c-subunit stoichiometries, but do not necessarily impair Na^+^ coupling [14]. Further experimental data will be required to clarify this question conclusively.

### ATP Synthase and c-rings of Human Pathogens as Potential Drug Targets

In recent years, the ATP synthase has come into focus as a novel drug target [41]. Of particular interest is the finding that the *M. tuberculosis* c-ring can be inhibited by diarylquinolines, a new class of antibiotic compounds against multi-drug resistant strains [42],[43]. Even in non-replicating or persister cells of *M. tuberculosis*, the PMF is obligatory for ATP homeostasis [44] and hence it is of medical interest to develop strategies to disrupt energy generation via the ATP synthase. This notion also holds true for *F. nucleatum*, which is a pervasive bacterium in dental plaque biofilms, and which is associated with periodontitis, one of the most common human infections [45]. The fact that the c-ring of the *F. nucleatum* ATP synthase is Na^+^-coupled and that its structure is significantly different from mitochondrial c-rings [13],[28] opens an opportunity for new selective drugs against this opportunistic human pathogen.

## Materials and Methods

### Molecular Modeling and Simulations

Molecular dynamics (MD) simulations and free-energy calculations were based on a homology model of the *F. nucleatum* c_11_ ring embedded in a phospholipid membrane (Figure S2) as described previously [46]. The Free-Energy Perturbation (FEP) method was employed in all ion-selectivity and pK_a_ calculations, as implemented in NAMD 2.7 [47]. Ab initio calculations were carried out with Gaussian09 (Frisch M.J. et al., Gaussian Inc., Wallingford CT, 2009) at the HF/6-31G* level. Further details see Text S1.

### *F. nucleatum* Culture Conditions

*F. nucleatum*, subsp. *nucleatum* ATCC25586 was grown anaerobically at 37°C in Columbia broth (Difco) supplemented with cysteine-hydrochloride (reductant) and resazurin as an oxygen indicator (pH 7.5). Cells were routinely grown in either anaerobic N_2_/H_2_/CO_2_ (90∶5∶5)-gassed Hungate tubes or in 1 l Schott bottles. For further details see Text S1.

### Preparation of *F. nucleatum* Inverted Membrane Vesicles

Cells grown to mid-exponential phase were passed three times through a French press cell (20,000 psi) and the emulsion was centrifuged (8,000 *g* for 10 min) to remove cell debris. Inverted membrane vesicles were collected by ultracentrifugation, washed twice, and adjusted to a final protein concentration of 20–30 mg/ml. For further details see Text S1.

### Solubilization, Purification, and Biochemical Analysis of the *F. nucleatum* F_1_F_o_-ATP Synthase

Inverted membrane vesicles were solubilized in reducing buffer (pH 7.5) using 2% (w/v) dodecylmaltoside, 10% (v/v) glycerol, and a protease inhibitor cocktail at 4°C for 1 h. The solubilizate was further purified by anion exchange chromatography and the ATP synthase-containing fractions were concentrated by ultrafiltration. The sample was further purified by gel filtration, desalted, and analyzed by SDS-PAGE. The identification of the F_1_F_o_-ATP synthase subunits was further confirmed by mass spectrometry (MALDI TOF/TOF MS). ATP hydrolysis activity was measured using the spectrophotometric ATP-regenerating assay or via the release of P_i_. ATP-driven proton translocation was determined by the quenching of acridine orange (AO), as described previously [48] with some modifications as described in Text S1. ATP synthesis in inverted membrane vesicles was determined via the standard luciferin-luciferase system, monitoring the light emitted with a chemiluminometer (FB 12 luminometer; Berthold) at 37°C, as described before [49]. For further details see Text S1.

### Production, Purification, Biochemical Analysis, and Crystallization of the *F. nucleatum* c-ring

The *F. nucleatum* c_11_ ring was produced in a heterologous hybrid ATP synthase expression system [50] forming the *I. tartaricus* ATP synthase (F_1_ab_2_) and the *F. nucleatum* c_11_ ring. The purification of the c-ring was performed by a procedure as described in Text S1. NCD-4 labeling experiments were performed as previously [24] with slight modifications described in Text S1. Crystallization of the *F. nucleatum* c_11_ ring was performed by vapor diffusion in hanging drops at 18°C. The crystals were flash-frozen in liquid nitrogen. For further details see Text S1.

### Data Collection, Structure Determination, and Data Deposition

Data to 2.2 Å (pH 5.3) and 2.64 Å (pH 8.7) were collected at the Max-Planck/Novartis beamline X10SA (PXII) of the Swiss Light Source (SLS, Villigen, Switzerland) and the beamline ID23.2 of the European Synchrotron Radiation Facility (ESRF, Grenoble, France), respectively. Data processing and structure modeling was performed as described in Text S1. The atomic coordinates and structure factors of the *F. nucleatum* c_11_ ring at pH 5.3 and pH 8.7 were deposited in the Protein Data Bank, under the accession numbers 3ZK1, and 3ZK2, respectively.

## Supporting Information

Figure S1 **Alignment of c-subunit sequences from F-ATP synthases of selected species.** The individual sequences were aligned according to their cytoplasmic loop region shown in bold. The single c-subunits form α-helical hairpins, the N- and C-terminal α-helices are highlighted in gray. The type of ion coordination (Na^+^ or H^+^) is indicated on the right side, the question marks in color indicate the assumed type of coordinated ion. The residues involved in ion coordination [6],[39] are highlighted in colors and the glycine-motif (GxGxGxGxG) is shown in bold. Species names: *F. nucleatum* subsp. *nucleatum* (numbering), *Ruminococcus albus*, *Thermotoga maritima*, *Eubacterium siraeum* DSM 15702, *Butyrivibrio proteoclasticus* B316, *Stomatobaculum longum*, *Oribacterium* sp. *oral* taxon 078 str. F0262, *Burkholderia pseudomallei*, *Azotobacter vinelandii*, *Gluconobacter oxydans*, *I. tartaricus*, *Propionigenium modestum*, *Clostridium paradoxum*, *Acetobacterium woodii*, *Mycoplasma genitalium*, *S. pneumoniae*, *M. tuberculosis*, *Aquifex aeolicus*, *Gloeobacter violaceus* PCC 7421, *Synechocystis* sp. strain PCC 6803, *Synoechococcus* sp. strain PCC 6716, *Arthrospira* sp. strain PCC 9438, *Spinacia oleracea* (chloroplast), *Bacillus* sp. strain PS3, *Caldalkalibacillus thermarum* TA2.A1 (*Bacillus* sp. strain TA2.A1), *Bacillus pseudofirmus* OF4, *Saccharomyces cerevisiae* (mitochondria), *Bos taurus* (mitochondria), *Haemophilus influenza*, *E. coli*. The abbreviated phyla names are: *Fu*, *Fusobacteria*; *Fi*, *Firmicutes*; *Th*, *Thermotogae*; *Pr*, *Proteobacteria*; *Ac*, *Actinobacteria; Aq Aquificae*; *Cy*, *Cyanobacteria*; *Vi*, *Viridiplantae*, *As*, *Ascomycota*; *Ch*, *Chordata*.
(TIF)

Figure S2 **Molecular model of the** ***F. nucleatum*** **c-ring embedded in a lipid membrane.** (A) View along the membrane plane, with the cytoplasmic side of the protein (yellow) at the top. The lipid membrane consists of 237 1-palmitoyl-2-oleoyl-*sn*-glycero-3-phosphocholine (POPC) molecules (blue), including those plugging the inner pore of the c-ring. The solvent includes ∼18,000 water molecules (red) and 22 chloride ions (orange spheres), which counter the net charge of the protein and thus neutralize the system. (B) Cross-section of the model c-ring, highlighting the hairpin-like transmembrane topology of one of the c-subunits, as well as the location of the bound Na^+^ ions (purple spheres) and the asymmetric lipid plug.
(TIF)

Figure S3 **Alternative models of the ion-binding site in the** ***F. nucleatum*** **c-ring, in the H^+^ bound state.** Hydrogen atoms in non-polar groups are omitted for clarity. In our models of the proton-bound state, Na^+^ is replaced by H^+^, which binds to Glu65, while Glu32 remains also protonated. Note the hydrogen-bond donated by Ser66 reorients accordingly. The water molecule that coordinates the bound Na^+^ is either preserved—as in the model shown in Figure 3A—or removed. (A) Alternate configuration of the site when a water molecule is preserved in the H^+^ bound state, similarly populated to that shown in Figure 3A. (B) Predicted structure of the site when the H^+^ state does not include the water molecule included in the Na^+^ state.
(TIF)

Figure S4 **Effect of ionophores on the growth of** ***F. nucleatum*** **ATCC 25586 in batch culture.** (A) CCCP was added to a final concentration (f.c.) of 50 µM (○), 100 µM (□) and 200 µM (Δ). (B) 2,4-dinitrophenol (DNP) was added to a f.c. of 50 µM (○), 100 µM (□) and 200 µM (Δ). (C) 3,3′,4′,5-tetrachlorosalicylanilide (TCS) was added to a f.c. of 2 µM (○), 5 µM (□), and 10 µM (Δ). (D) Monensin was added to a f.c. of 1 µM (○), 5 µM (□), and 10 µM (Δ). (E) Amiloride was added to a f.c. of 50 µM (○), 100 µM (□), and 200 µM (Δ). (F) 5-(*N*-ethyl-*N*-isopropyl)amiloride (EIPA) was added to a f.c. of 50 µM (○), 100 µM (□), and 200 µM (Δ). Controls were grown with an equivalent volume of ethanol (•). The values plotted are the mean of three biological replicates and their standard errors.
(TIF)

Figure S5 **Effect of ATP synthase inhibitors on the growth of** ***F. nucleatum*** **ATCC 25586 in batch culture.** (A) DCCD was added to a final concentration of 100 µM (○), 200 µM (□), and 400 µM (Δ). (B) Tributyltin chloride (TBT-Cl) was added to a final concentration of 25 µM (○), 50 µM (□), and 150 µM (Δ). Controls were grown with an equivalent volume of ethanol (•). The values plotted are the mean of three biological replicates and their standard errors.
(TIF)

Figure S6 **ATP synthesis and ATP hydrolysis of** ***F. nucleatum*** **inverted membrane vesicles.** (A) Effect of external pH on the ATPase activity catalyzed by an F-type ATP synthase. The activity was measured in 50 mM MES-MOPS-Tris, 2 mM MgCl_2_ at 37°C by the ATP regenerating assay (250 µg membrane protein). 100% of ATPase activity corresponds to 0.09–0.15 units/mg protein (mg P) (1 unit = 1 µmol ATP hydrolyzed/min). (B) ATP synthesis (in nmol/min/mg of protein) in inverted membrane vesicles was energized by a valinomycin (2 µM)-induced potassium diffusion potential (100 mV) applied either in the absence (i) or in the presence (ii) of a chemical gradient of Na^+^. Effect of 5 µM monensin (iii) or 150 µM DCCD (iv) on ATP synthesis energized by a valinomycin-induced potassium diffusion potential, applied in presence of a chemical gradient of Na^+^. ATP synthesis with no added valinomycin in the absence (v) or in the presence (vi) of a chemical gradient of Na^+^. All inhibitors were preincubated with the inverted membrane vesicles for 10 min prior to the addition of valinomycin.
(TIF)

Figure S7 **Analysis of purified** ***F. nucleatum*** **ATP synthase by silver-stained 12.5% SDS-PAGE.** Lane 1, prestained molecular mass marker (ThermoScientific), indicated in kDa; lane 2, selected fraction (2.3 µg protein) from gel filtration column. The identity and molecular mass of the constituent protein subunits in kDa are indicated on the right. MALDI mass spectrometry analysis identified subunits α, β, γ, δ, and b (unpublished data). The bands for the c_11_ ring and the β-subunit overlap.
(TIF)

Figure S8 **Catalytic activity of the purified F_1_F_o_-ATP synthase from** ***F. nucleatum*** **.** (A) Hydrolytic activity of the ATP synthase in membranes, soluble fraction (DDM extracted) and purified protein over time (all stored at 4°C). (B) Effect of ATP on hydrolytic activity of the purified enzyme at 37°C. (C) Effect of MgCl_2_ on hydrolytic activity at 37°C. The ATPase activity was determined using the ATP regenerating assay (120–140 µg protein) in (A), (B), and the P_i_ assay (60–70 µg protein) in (C). 100% of activity corresponds to 0.15 units/mg of protein for membrane vesicles and 1–2 units/mg for the purified protein at pH 7.5. The ATP hydrolysis assay mixture contained 50 mM MOPS, 2 mM MgCl_2_ (pH 7.5). The values plotted are representative of two to three biological replicates; the statistical variance was less than 20%.
(TIF)

Figure S9 **Inhibition of the purified** ***F. nucleatum*** **ATP synthase by DCCD.** The purified protein (120–140 µg) was incubated at 25°C in 50 mM MOPS, 2 mM MgCl_2_, pH 7.5 for 20 min, with the DCCD concentrations indicated. The control sample was incubated with an equal amount of ethanol. The ATPase activity was quantified using the ATP-regenerating assay. 100% of activity corresponds to 1–2 µmol ATP/min/mg of protein. Values are representative of two to three separate experiments; the statistical variance was less than 20%.
(TIF)

Figure S10 **Silver-stained SDS-PAGE of the purified c_11_ ring from** ***F. nucleatum*** **.** Lane 1, 1 µg of c_11_ ring; lane 2, 1 µg of c-ring precipitated with 15% (w/v) trichloroacetic acid. The c-monomer (c_1_), c-dimer (c_2_), and c-oligomer (c_11_) are indicated on the right. A molecular weight marker (M, PageRuler Unstained Protein Ladder, Fermentas) in kDa is given on the left.
(TIF)

Figure S11 **Determination of the c-subunit mass from the isolated c-ring of** ***F. nucleatum*** **by MALDI-MS.** The theoretical mass of the c-monomer is 8,862.94 Da (unformylated) and 8,890.94 Da (formylated); the theoretical mass of the formylated, single-oxidized c-monomer is 8,906.94 Da, and 8,922.94 Da for the double-oxidized form.
(TIF)

Figure S12 **Long-term kinetics of NCD-4 modification of detergent-solubilized c-rings from** ***F. nucleatum*** **, without the addition of Na^+^.** A 27 µg c-ring sample in 0.5 M MES buffer pH 5.7 containing 1.5% (w/v) n-octyl-β-D-glycoside was monitored in a fluorescence spectrophotometer (λ_ex_ = 342 nm, λ_em_ = 452 nm) at selected time points for a total of 120 min. The reaction was initiated by the addition of 100 µM NCD-4 (in 10% (w/v) β-dodecyl-maltoside) at time point 0. The arrow indicates the time point at which the NCD-4 labeling of the c-ring was stopped in the experiment reported in Figure 6, by addition of 15 mM NaCl.
(TIF)

Figure S13 **SDS-PAGE of the c-ring and the c-monomer from** ***F. nucleatum*** **after reaction with NCD-4.** The NCD-4 modified *F. nucleatum* c_11_ ring was loaded on a 13.2% SDS-polyacrylamide gel. The same sample was also precipitated using 15% (w/v) trichloroacetic acid (+TCA), resulting in monomeric c-subunits (as indicated). (A) Silver-stained SDS-polyacrylamide gel, showing a shift in the apparent molecular mass of c_11_ rings, c_1_ monomers, and c_2_ dimers, after binding of NCD-4 (thin dashed lines). Unmodified (non NCD-4 treated) samples were added for comparison, showing a slightly faster migration. (B) UV-light exposed SDS-polyacrylamide gel, showing the fluorescence of the NCD-4 modified c_11_ rings and c_1_ monomers. Two molecular mass markers (M1, PageRuler Unstained Protein Ladder, Fermentas and M2, PageRuler Prestained Protein Ladder, Fermentas) are indicated in kDa on the left side of both gels.
(TIF)

Figure S14 **Comparison of the experimental and predicted structures of the** ***F. nucleatum*** **c-ring.** The experimental structure (blue cartoons) is that obtained by X-ray crystallography at pH 5.3. The predicted structure (red cartoons) is a time-average calculated from the simulation of model A (Figure 1A), which was generated by homology with the c-ring of *I. tartaricus*. The RMS difference between the backbone conformations is 0.74 Å. The c-ring is viewed (A) from the plane of the membrane, and (B) along the membrane perpendicular.
(TIF)

Table S1 **Purification of the** ***F. nucleatum*** **F_1_F_o_-ATP synthase.**
(DOC)

Table S2 **Collection of X-ray diffracion data and refinement statistics.**
(DOC)

Text S1 **Supporting materials and methods, and references.**
(DOC)

## Abbreviations

CCCP: carbonyl cyanide m-chlorophenyl hydrazine
NCD-4: *N*-cyclohexyl-*N*′-(4-(dimethylamino)-α-naphthyl)-carbodiimide
PMF: proton-motive force
SMF: sodium-motive force
DCCD: *N*,*N*′-dicyclohexylcarbodiimide
