# Supplementary figures and images for: A New Type of Na+-Driven ATP Synthase Membrane Rotor with a Two-Carboxylate Ion-Coupling Motif
Source: PLoS Biol. 2013 Jun 25;11(6):e1001596. doi: 10.1371/journal.pbio.1001596 (PMC3692424; doi:10.1371/journal.pbio.1001596)

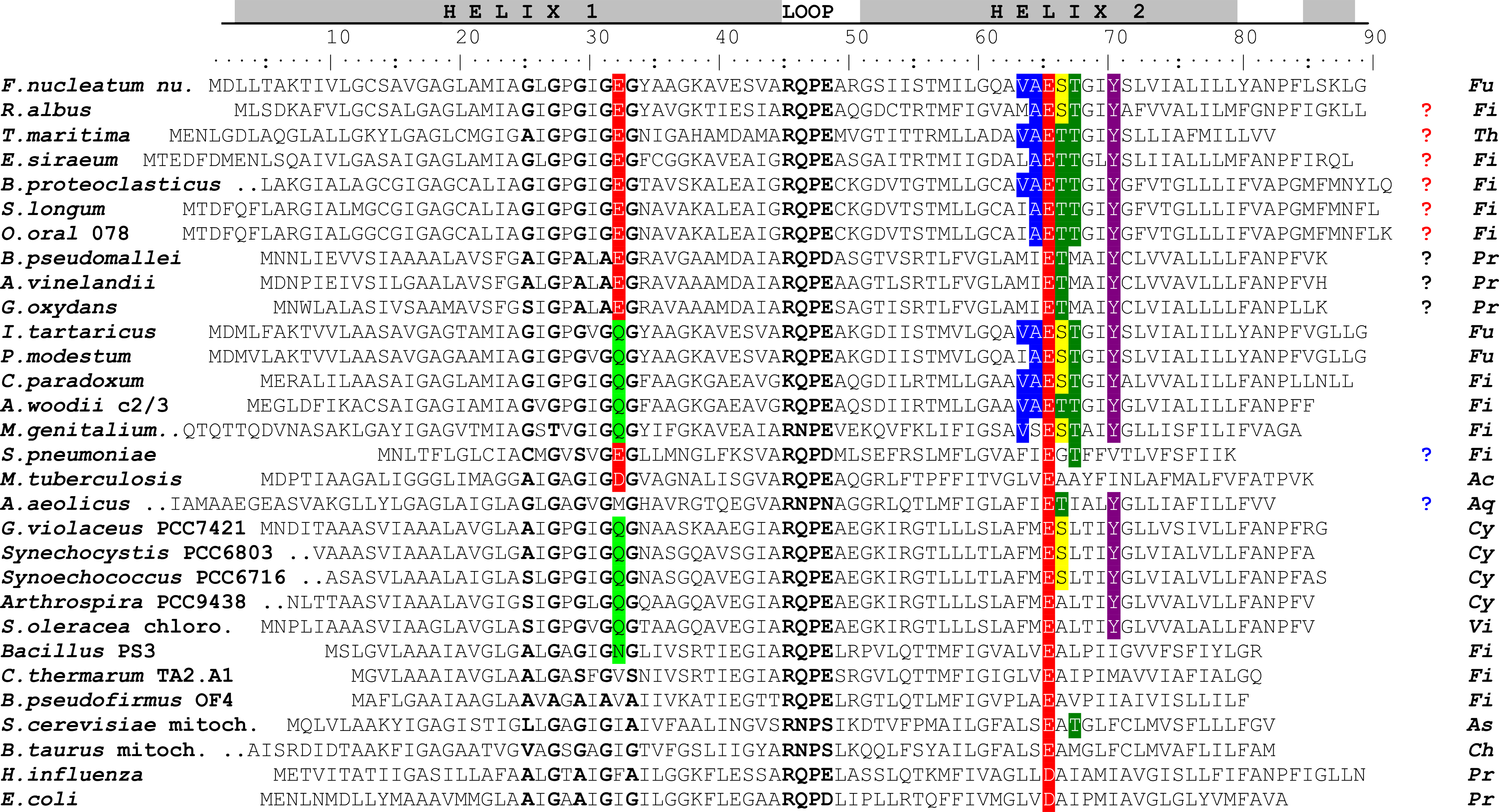

Supplement: Figure S1 — Alignment of c-subunit sequences from F-ATP synthases of selected species. The individual sequences were aligned according to their cytoplasmic loop region shown in bold. The single c-subunits form α-helical hairpins, the N- and C-terminal α-helices are highlighted in gray. The type of ion coordination (Na+ or H+) is indicated on the right side, the question marks in color indicate the assumed type of coordinated ion. The residues involved in ion coordination [6],[39] are highlighted in colors and the glycine-motif (GxGxGxGxG) is shown in bold. Species names: F. nucleatum subsp. nucleatum (numbering), Ruminococcus albus, Thermotoga maritima, Eubacterium siraeum DSM 15702, Butyrivibrio proteoclasticus B316, Stomatobaculum longum, Oribacterium sp. oral taxon 078 str. F0262, Burkholderia pseudomallei, Azotobacter vinelandii, Gluconobacter oxydans, I. tartaricus, Propionigenium modestum, Clostridium paradoxum, Acetobacterium woodii, Mycoplasma genitalium, S. pneumoniae, M. tuberculosis, Aquifex aeolicus, Gloeobacter violaceus PCC 7421, Synechocystis sp. strain PCC 6803, Synoechococcus sp. strain PCC 6716, Arthrospira sp. strain PCC 9438, Spinacia oleracea (chloroplast), Bacillus sp. strain PS3, Caldalkalibacillus thermarum TA2.A1 (Bacillus sp. strain TA2.A1), Bacillus pseudofirmus OF4, Saccharomyces cerevisiae (mitochondria), Bos taurus (mitochondria), Haemophilus influenza, E. coli. The abbreviated phyla names are: Fu, Fusobacteria; Fi, Firmicutes; Th, Thermotogae; Pr, Proteobacteria; Ac, Actinobacteria; Aq Aquificae; Cy, Cyanobacteria; Vi, Viridiplantae, As, Ascomycota; Ch, Chordata. (TIF) [file pbio.1001596.s001.tif]

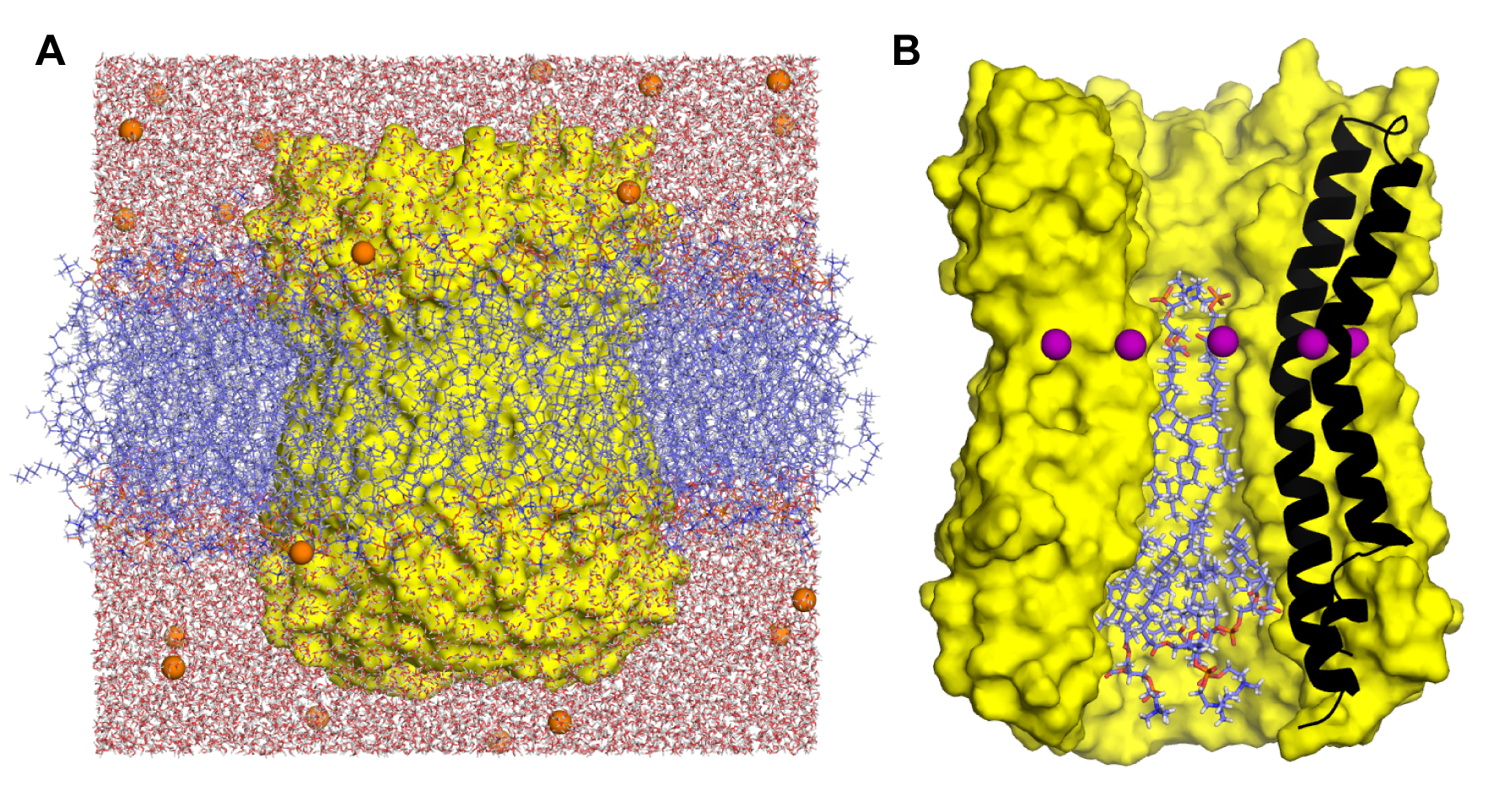

Supplement: Figure S2 — Molecular model of the F. nucleatum c-ring embedded in a lipid membrane. (A) View along the membrane plane, with the cytoplasmic side of the protein (yellow) at the top. The lipid membrane consists of 237 1-palmitoyl-2-oleoyl-sn-glycero-3-phosphocholine (POPC) molecules (blue), including those plugging the inner pore of the c-ring. The solvent includes ∼18,000 water molecules (red) and 22 chloride ions (orange spheres), which counter the net charge of the protein and thus neutralize the system. (B) Cross-section of the model c-ring, highlighting the hairpin-like transmembrane topology of one of the c-subunits, as well as the location of the bound Na+ ions (purple spheres) and the asymmetric lipid plug. (TIF) [file pbio.1001596.s002.tif]

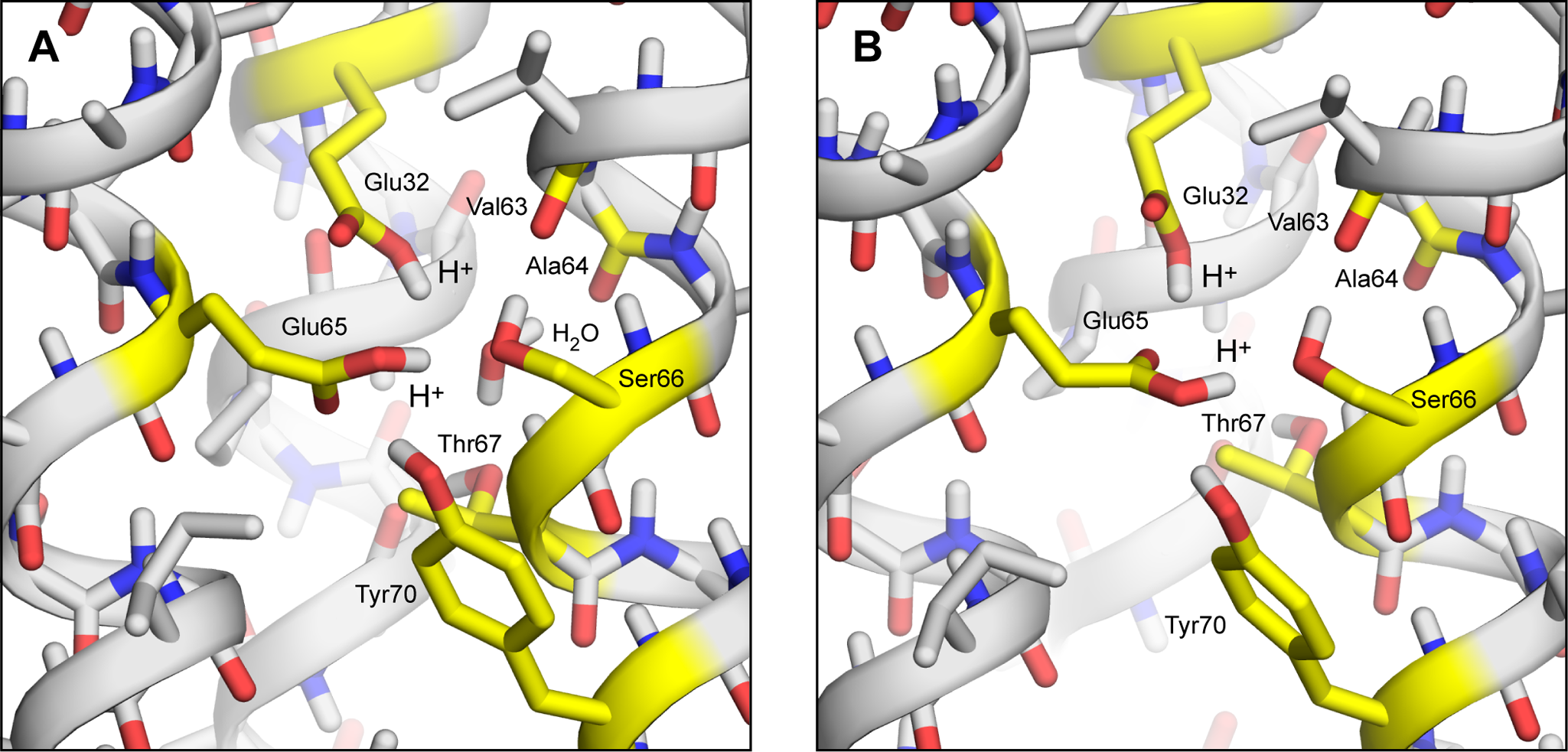

Supplement: Figure S3 — Alternative models of the ion-binding site in the F. nucleatum c-ring, in the H+ bound state. Hydrogen atoms in non-polar groups are omitted for clarity. In our models of the proton-bound state, Na+ is replaced by H+, which binds to Glu65, while Glu32 remains also protonated. Note the hydrogen-bond donated by Ser66 reorients accordingly. The water molecule that coordinates the bound Na+ is either preserved—as in the model shown in Figure 3A—or removed. (A) Alternate configuration of the site when a water molecule is preserved in the H+ bound state, similarly populated to that shown in Figure 3A. (B) Predicted structure of the site when the H+ state does not include the water molecule included in the Na+ state. (TIF) [file pbio.1001596.s003.tif]

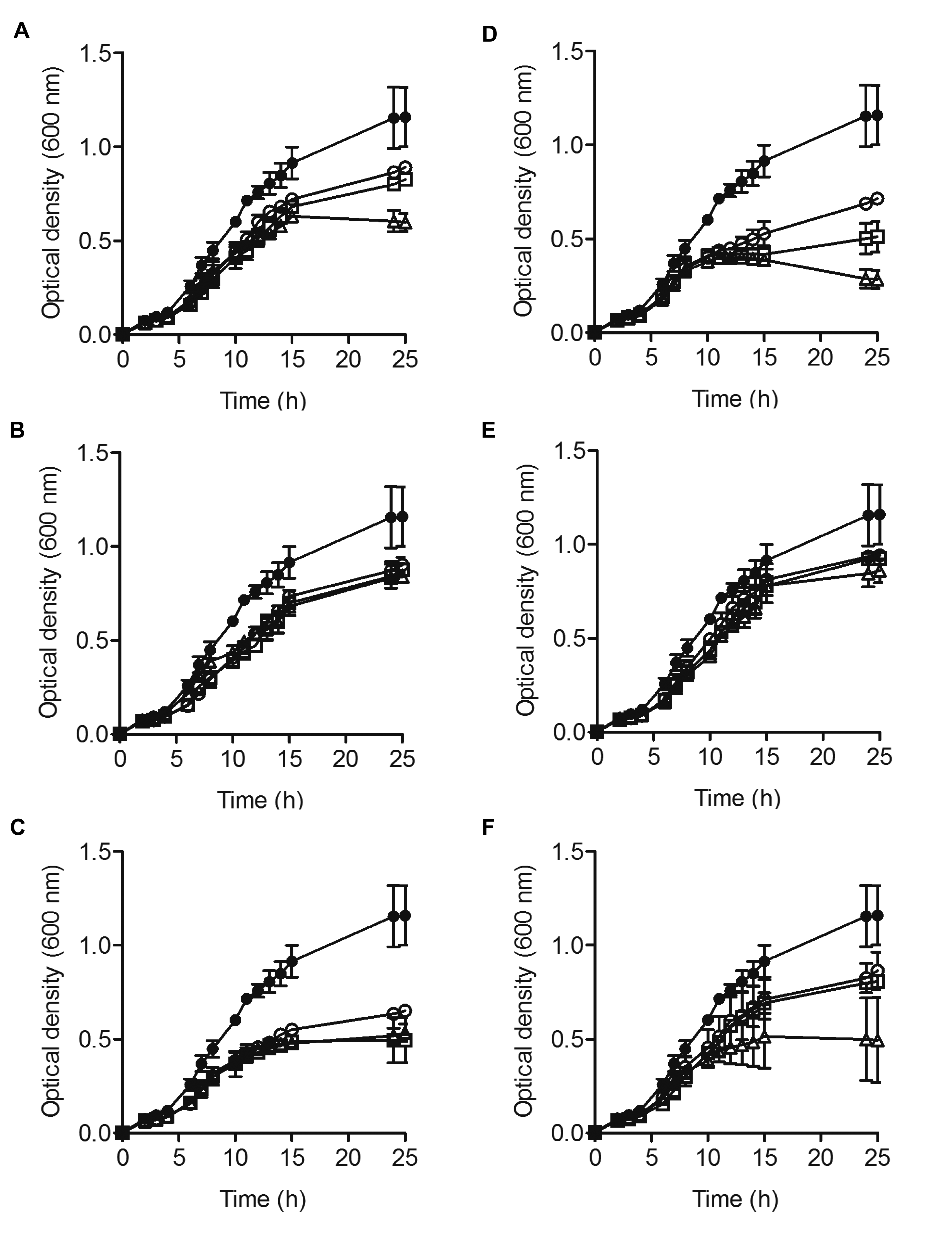

Supplement: Figure S4 — Effect of ionophores on the growth of F. nucleatum ATCC 25586 in batch culture. (A) CCCP was added to a final concentration (f.c.) of 50 µM (○), 100 µM (□) and 200 µM (Δ). (B) 2,4-dinitrophenol (DNP) was added to a f.c. of 50 µM (○), 100 µM (□) and 200 µM (Δ). (C) 3,3′,4′,5-tetrachlorosalicylanilide (TCS) was added to a f.c. of 2 µM (○), 5 µM (□), and 10 µM (Δ). (D) Monensin was added to a f.c. of 1 µM (○), 5 µM (□), and 10 µM (Δ). (E) Amiloride was added to a f.c. of 50 µM (○), 100 µM (□), and 200 µM (Δ). (F) 5-(N-ethyl-N-isopropyl)amiloride (EIPA) was added to a f.c. of 50 µM (○), 100 µM (□), and 200 µM (Δ). Controls were grown with an equivalent volume of ethanol (•). The values plotted are the mean of three biological replicates and their standard errors. (TIF) [file pbio.1001596.s004.tif]

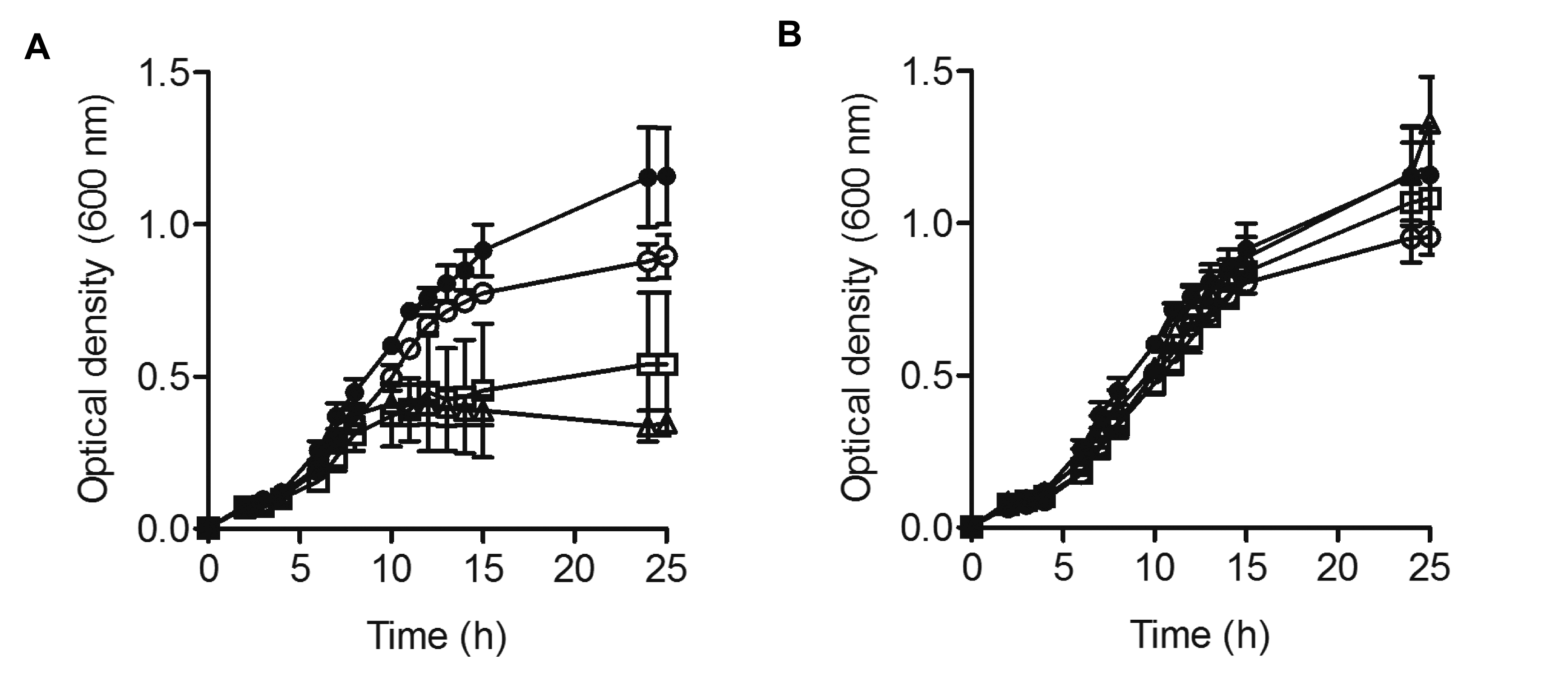

Supplement: Figure S5 — Effect of ATP synthase inhibitors on the growth of F. nucleatum ATCC 25586 in batch culture. (A) DCCD was added to a final concentration of 100 µM (○), 200 µM (□), and 400 µM (Δ). (B) Tributyltin chloride (TBT-Cl) was added to a final concentration of 25 µM (○), 50 µM (□), and 150 µM (Δ). Controls were grown with an equivalent volume of ethanol (•). The values plotted are the mean of three biological replicates and their standard errors. (TIF) [file pbio.1001596.s005.tif]

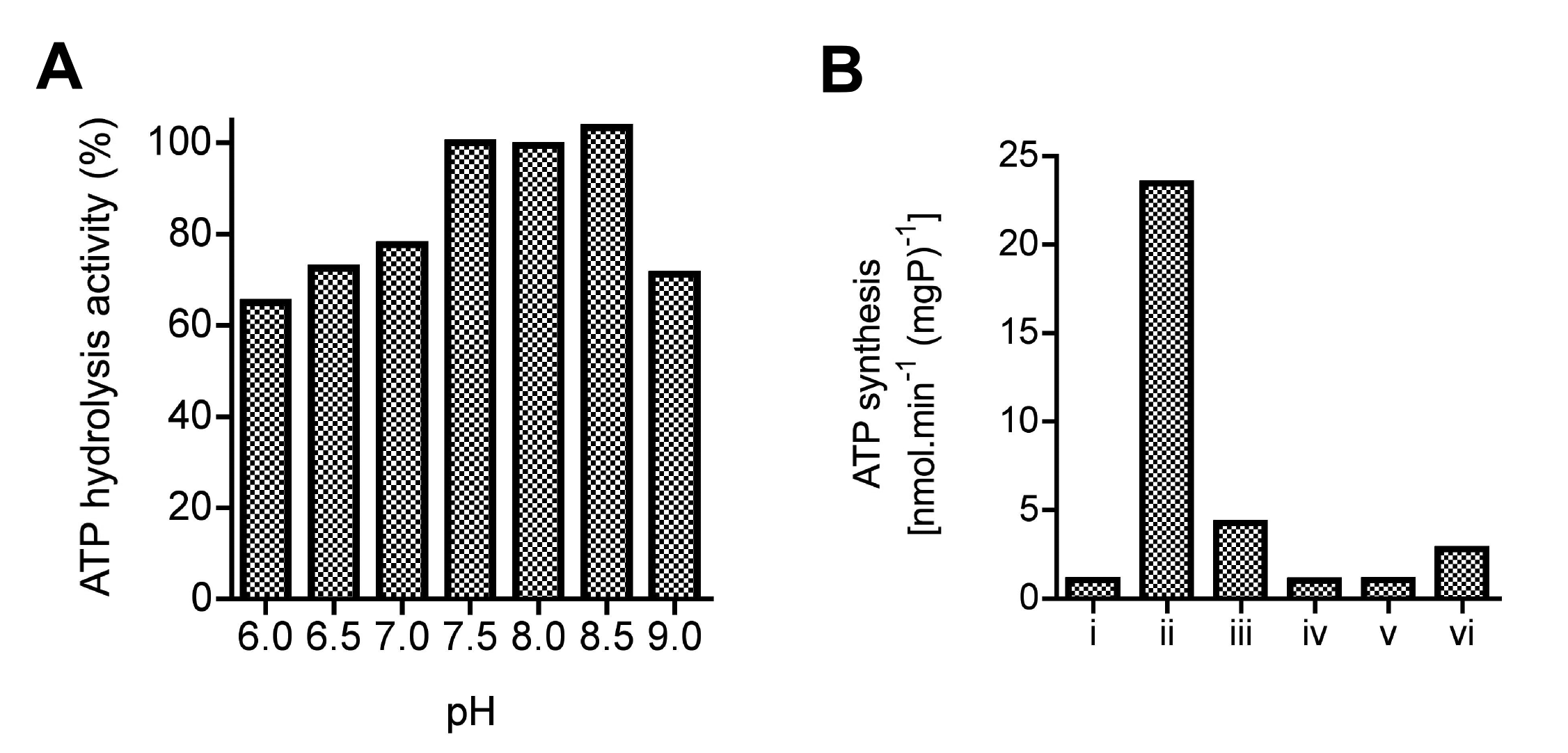

Supplement: Figure S6 — ATP synthesis and ATP hydrolysis of F. nucleatum inverted membrane vesicles. (A) Effect of external pH on the ATPase activity catalyzed by an F-type ATP synthase. The activity was measured in 50 mM MES-MOPS-Tris, 2 mM MgCl2 at 37°C by the ATP regenerating assay (250 µg membrane protein). 100% of ATPase activity corresponds to 0.09–0.15 units/mg protein (mg P) (1 unit = 1 µmol ATP hydrolyzed/min). (B) ATP synthesis (in nmol/min/mg of protein) in inverted membrane vesicles was energized by a valinomycin (2 µM)-induced potassium diffusion potential (100 mV) applied either in the absence (i) or in the presence (ii) of a chemical gradient of Na+. Effect of 5 µM monensin (iii) or 150 µM DCCD (iv) on ATP synthesis energized by a valinomycin-induced potassium diffusion potential, applied in presence of a chemical gradient of Na+. ATP synthesis with no added valinomycin in the absence (v) or in the presence (vi) of a chemical gradient of Na+. All inhibitors were preincubated with the inverted membrane vesicles for 10 min prior to the addition of valinomycin. (TIF) [file pbio.1001596.s006.tif]

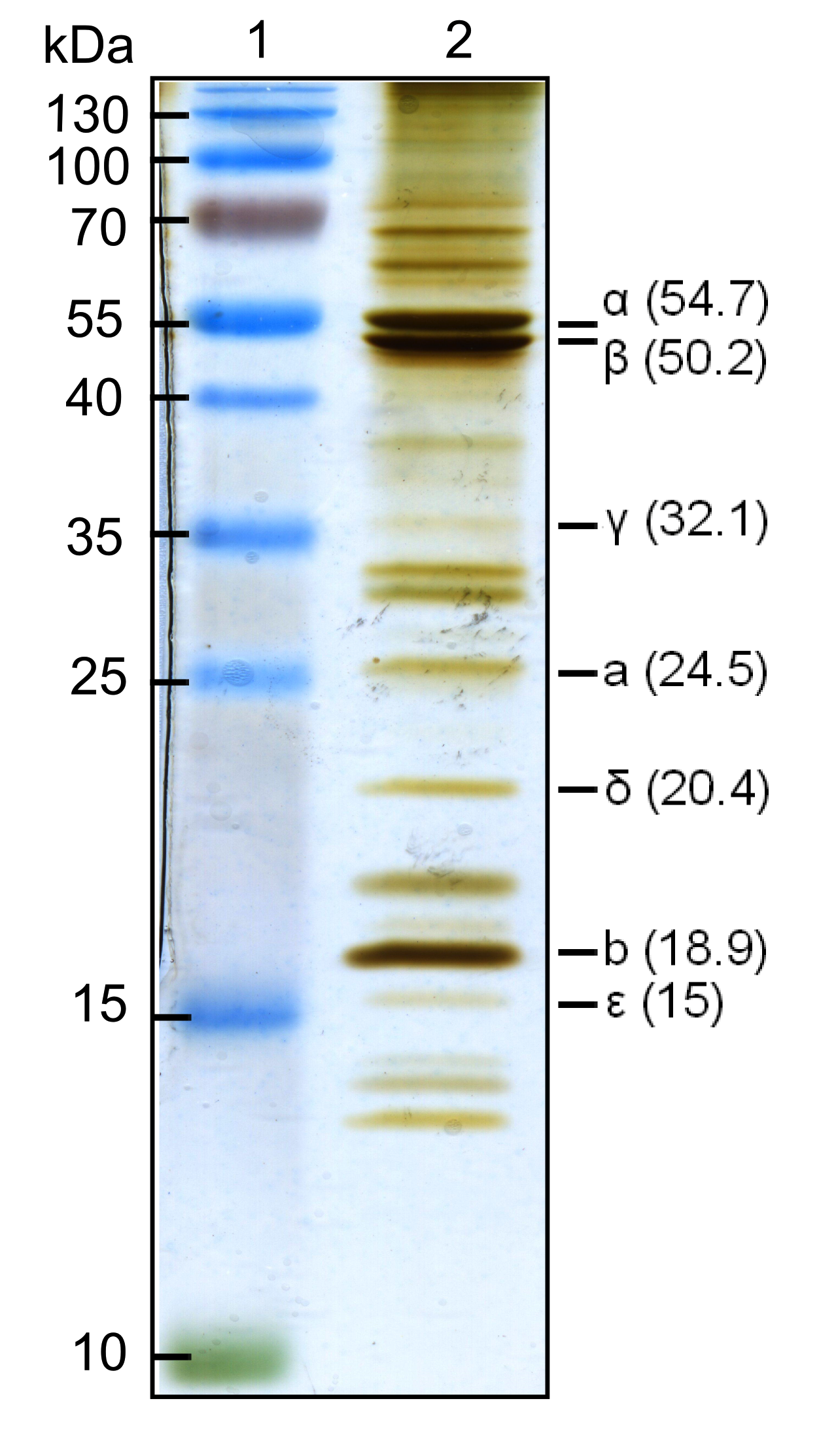

Supplement: Figure S7 — Analysis of purified F. nucleatum ATP synthase by silver-stained 12.5% SDS-PAGE. Lane 1, prestained molecular mass marker (ThermoScientific), indicated in kDa; lane 2, selected fraction (2.3 µg protein) from gel filtration column. The identity and molecular mass of the constituent protein subunits in kDa are indicated on the right. MALDI mass spectrometry analysis identified subunits α, β, γ, δ, and b (unpublished data). The bands for the c11 ring and the β-subunit overlap. (TIF) [file pbio.1001596.s007.tif]

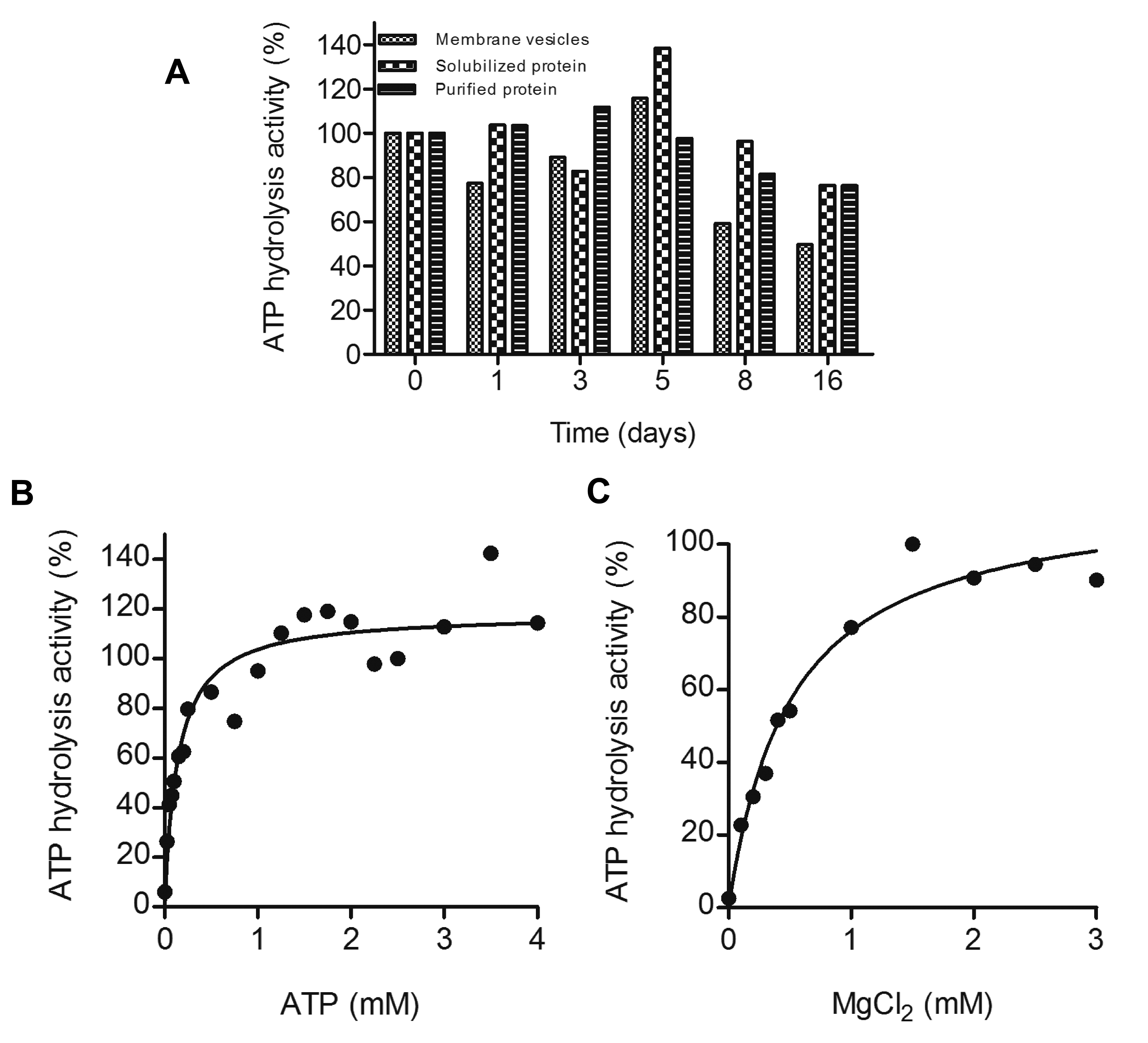

Supplement: Figure S8 — Catalytic activity of the purified F1Fo-ATP synthase from F. nucleatum . (A) Hydrolytic activity of the ATP synthase in membranes, soluble fraction (DDM extracted) and purified protein over time (all stored at 4°C). (B) Effect of ATP on hydrolytic activity of the purified enzyme at 37°C. (C) Effect of MgCl2 on hydrolytic activity at 37°C. The ATPase activity was determined using the ATP regenerating assay (120–140 µg protein) in (A), (B), and the Pi assay (60–70 µg protein) in (C). 100% of activity corresponds to 0.15 units/mg of protein for membrane vesicles and 1–2 units/mg for the purified protein at pH 7.5. The ATP hydrolysis assay mixture contained 50 mM MOPS, 2 mM MgCl2 (pH 7.5). The values plotted are representative of two to three biological replicates; the statistical variance was less than 20%. (TIF) [file pbio.1001596.s008.tif]

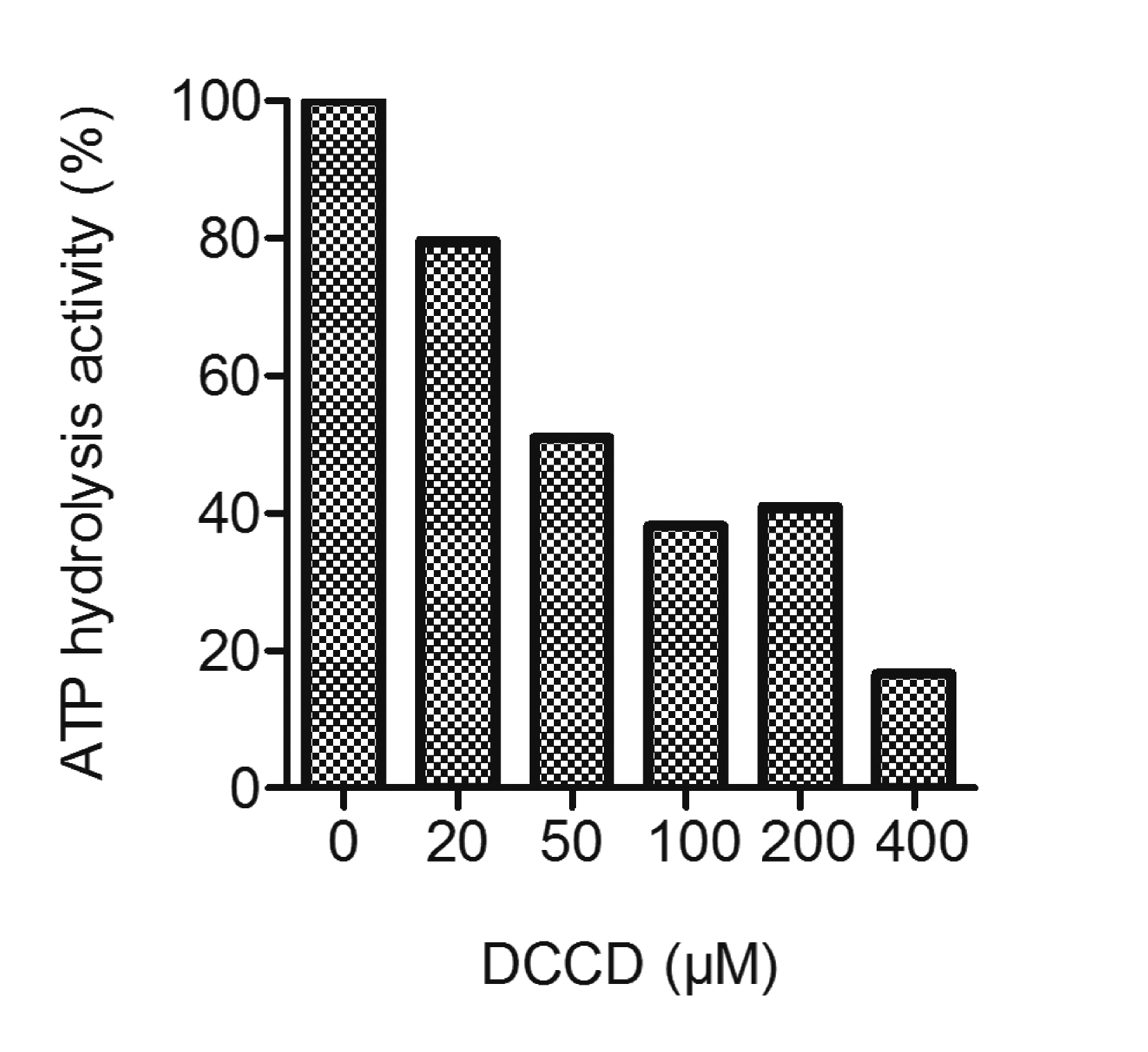

Supplement: Figure S9 — Inhibition of the purified F. nucleatum ATP synthase by DCCD. The purified protein (120–140 µg) was incubated at 25°C in 50 mM MOPS, 2 mM MgCl2, pH 7.5 for 20 min, with the DCCD concentrations indicated. The control sample was incubated with an equal amount of ethanol. The ATPase activity was quantified using the ATP-regenerating assay. 100% of activity corresponds to 1–2 µmol ATP/min/mg of protein. Values are representative of two to three separate experiments; the statistical variance was less than 20%. (TIF) [file pbio.1001596.s009.tif]

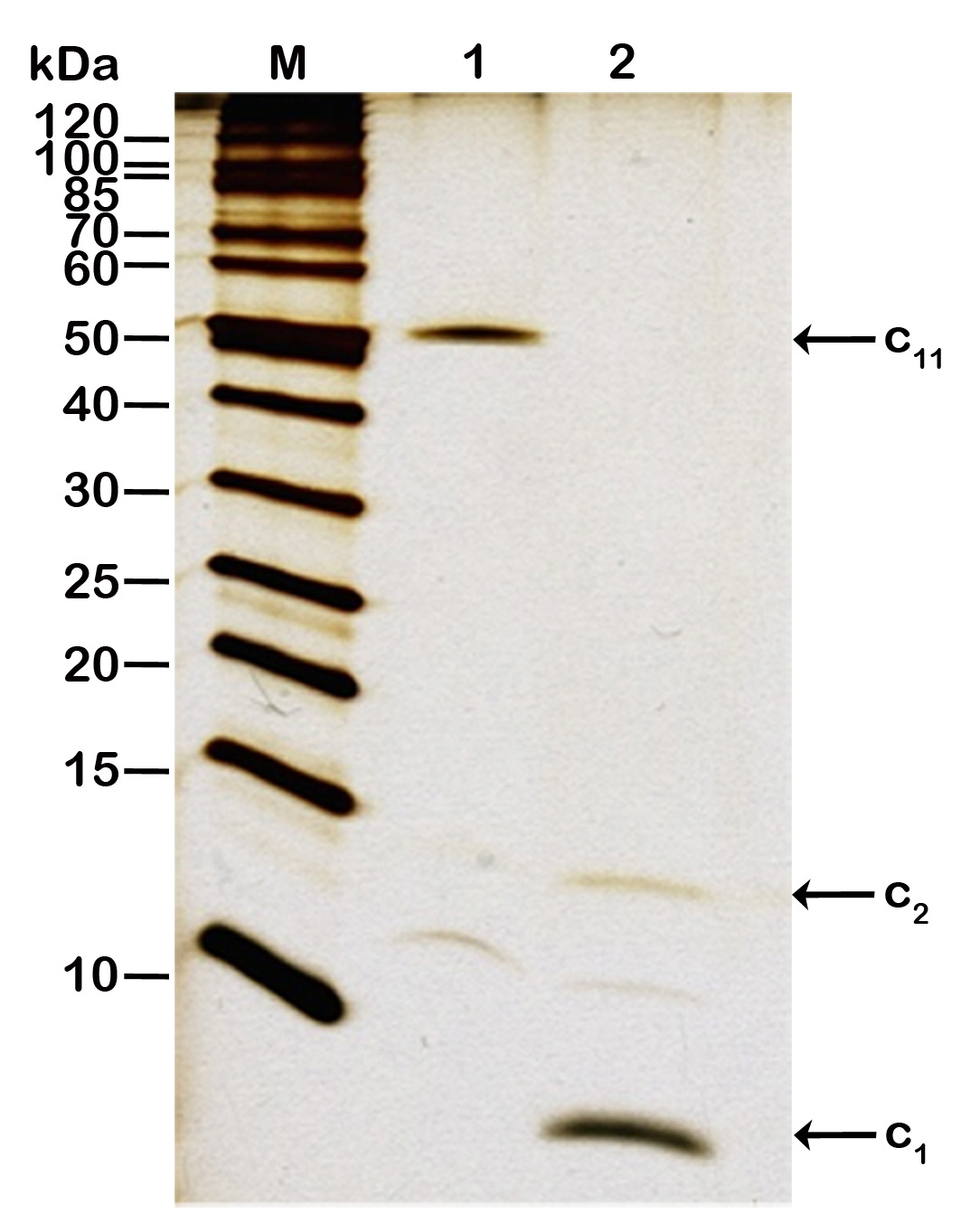

Supplement: Figure S10 — Silver-stained SDS-PAGE of the purified c11 ring from F. nucleatum . Lane 1, 1 µg of c11 ring; lane 2, 1 µg of c-ring precipitated with 15% (w/v) trichloroacetic acid. The c-monomer (c1), c-dimer (c2), and c-oligomer (c11) are indicated on the right. A molecular weight marker (M, PageRuler Unstained Protein Ladder, Fermentas) in kDa is given on the left. (TIF) [file pbio.1001596.s010.tif]

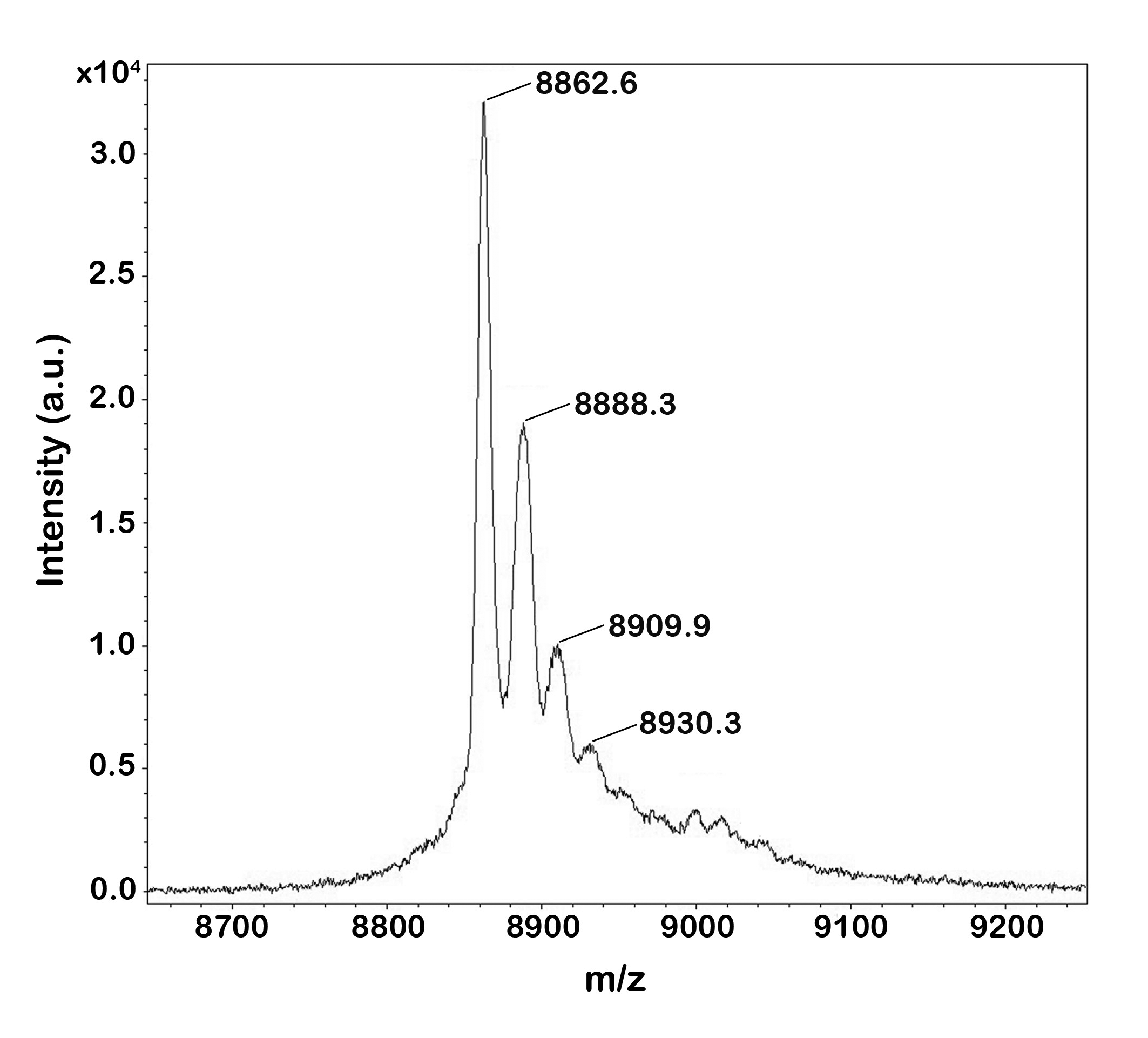

Supplement: Figure S11 — Determination of the c-subunit mass from the isolated c-ring of F. nucleatum by MALDI-MS. The theoretical mass of the c-monomer is 8,862.94 Da (unformylated) and 8,890.94 Da (formylated); the theoretical mass of the formylated, single-oxidized c-monomer is 8,906.94 Da, and 8,922.94 Da for the double-oxidized form. (TIF) [file pbio.1001596.s011.tif]

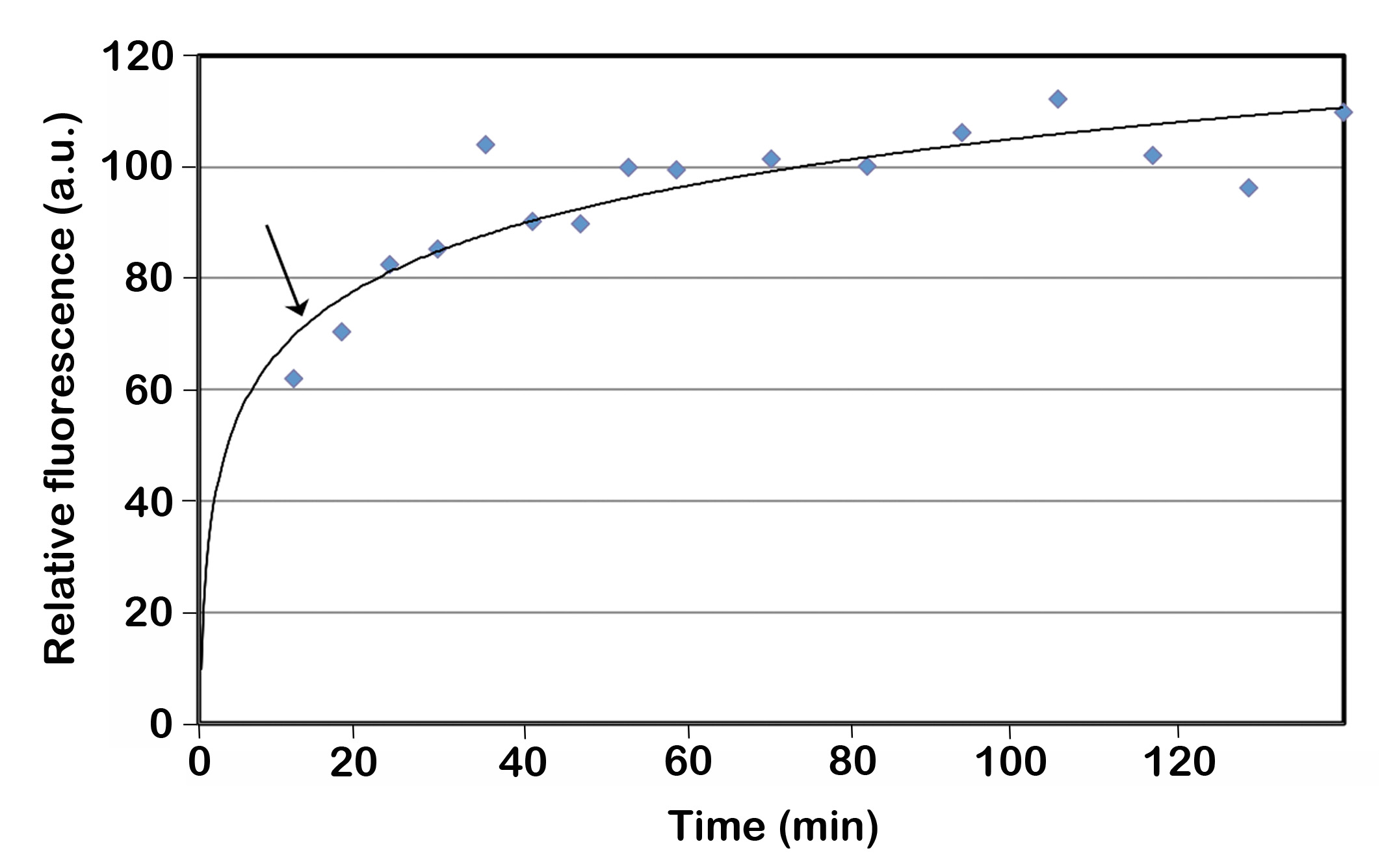

Supplement: Figure S12 — Long-term kinetics of NCD-4 modification of detergent-solubilized c-rings from F. nucleatum , without the addition of Na+. A 27 µg c-ring sample in 0.5 M MES buffer pH 5.7 containing 1.5% (w/v) n-octyl-β-D-glycoside was monitored in a fluorescence spectrophotometer (λex = 342 nm, λem = 452 nm) at selected time points for a total of 120 min. The reaction was initiated by the addition of 100 µM NCD-4 (in 10% (w/v) β-dodecyl-maltoside) at time point 0. The arrow indicates the time point at which the NCD-4 labeling of the c-ring was stopped in the experiment reported in Figure 6, by addition of 15 mM NaCl. (TIF) [file pbio.1001596.s012.tif]

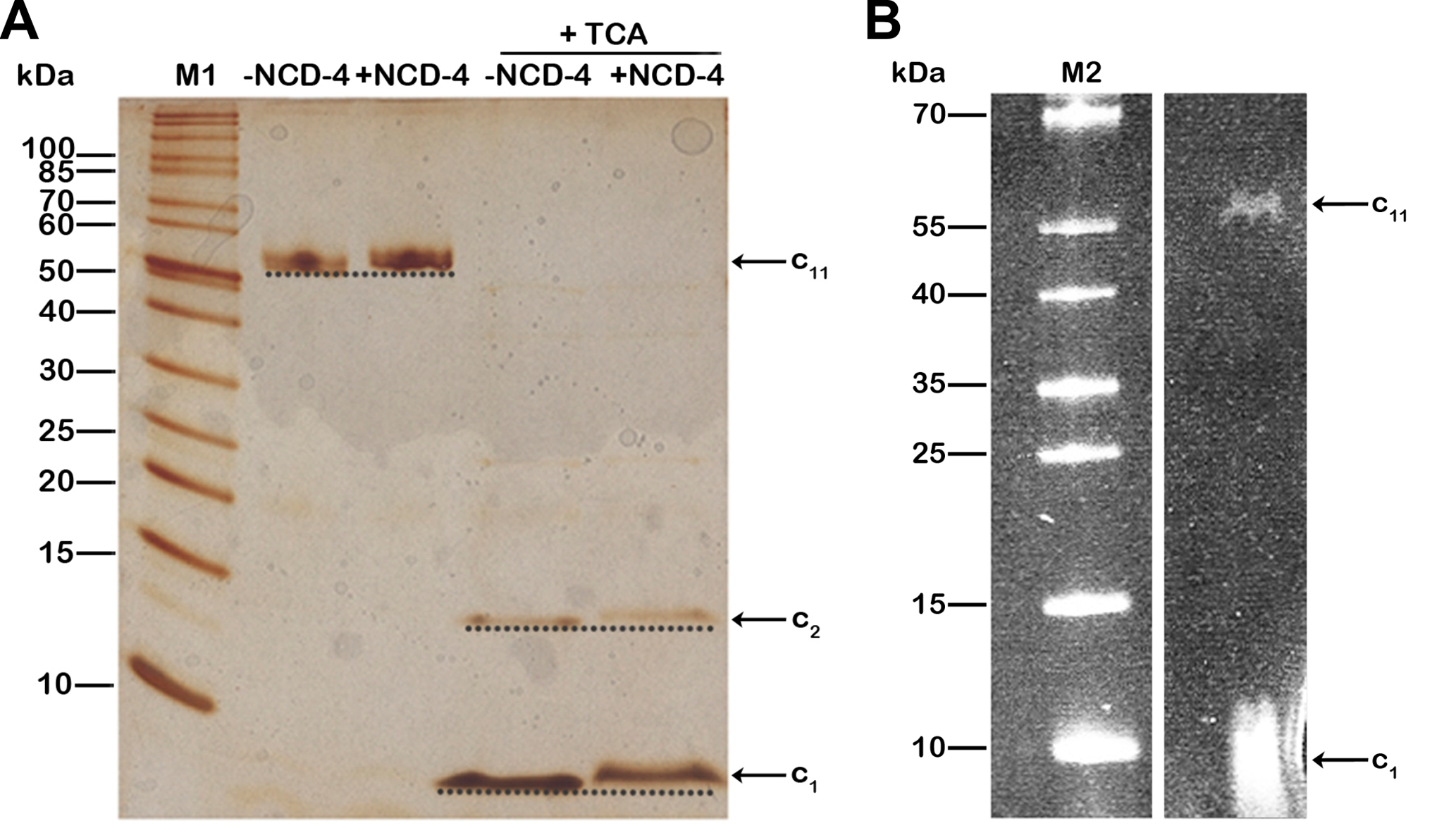

Supplement: Figure S13 — SDS-PAGE of the c-ring and the c-monomer from F. nucleatum after reaction with NCD-4. The NCD-4 modified F. nucleatum c11 ring was loaded on a 13.2% SDS-polyacrylamide gel. The same sample was also precipitated using 15% (w/v) trichloroacetic acid (+TCA), resulting in monomeric c-subunits (as indicated). (A) Silver-stained SDS-polyacrylamide gel, showing a shift in the apparent molecular mass of c11 rings, c1 monomers, and c2 dimers, after binding of NCD-4 (thin dashed lines). Unmodified (non NCD-4 treated) samples were added for comparison, showing a slightly faster migration. (B) UV-light exposed SDS-polyacrylamide gel, showing the fluorescence of the NCD-4 modified c11 rings and c1 monomers. Two molecular mass markers (M1, PageRuler Unstained Protein Ladder, Fermentas and M2, PageRuler Prestained Protein Ladder, Fermentas) are indicated in kDa on the left side of both gels. (TIF) [file pbio.1001596.s013.tif]

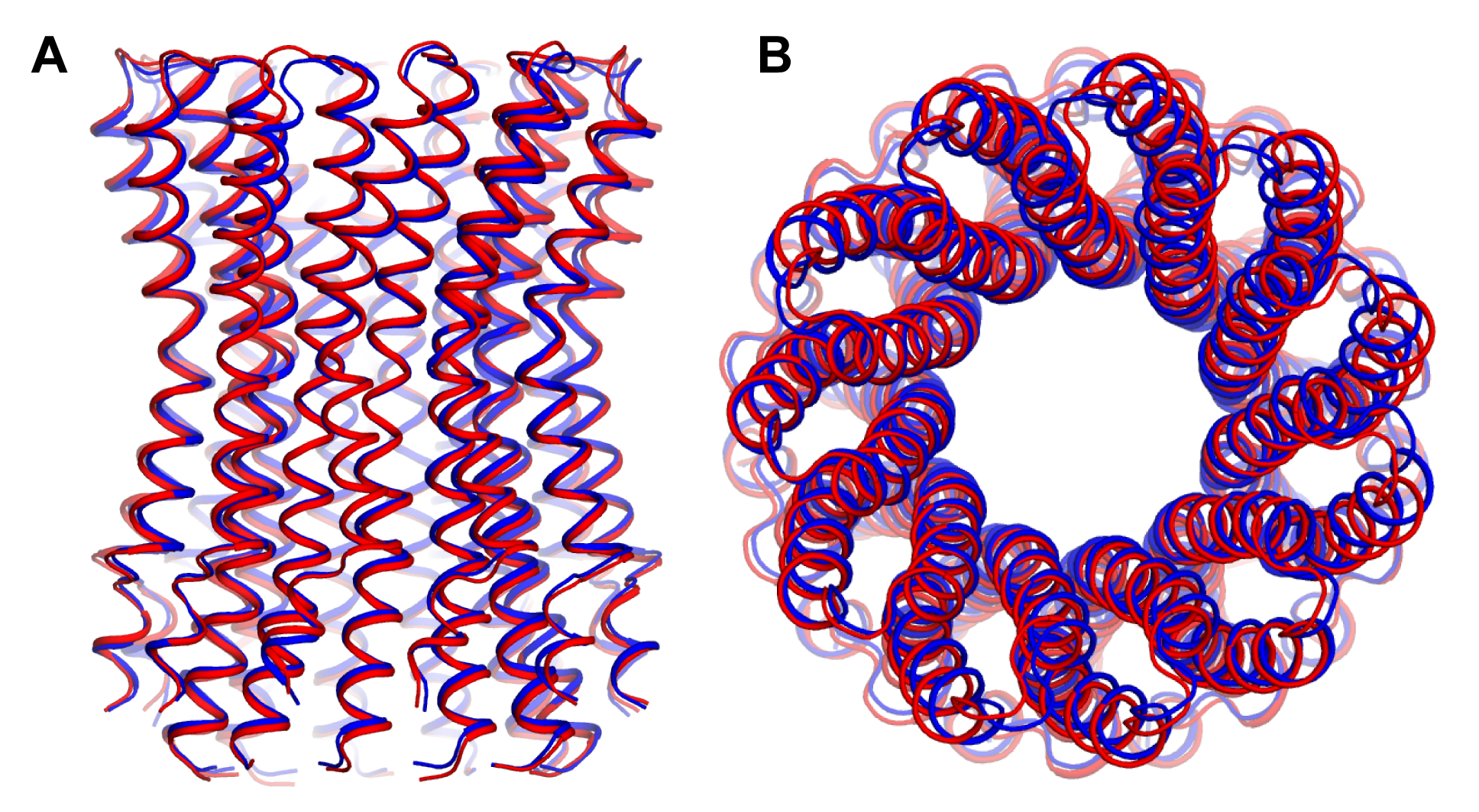

Supplement: Figure S14 — Comparison of the experimental and predicted structures of the F. nucleatum c-ring. The experimental structure (blue cartoons) is that obtained by X-ray crystallography at pH 5.3. The predicted structure (red cartoons) is a time-average calculated from the simulation of model A (Figure 1A), which was generated by homology with the c-ring of I. tartaricus. The RMS difference between the backbone conformations is 0.74 Å. The c-ring is viewed (A) from the plane of the membrane, and (B) along the membrane perpendicular. (TIF) [file pbio.1001596.s014.tif]
