# Supplementary material for: A New Type of Na+-Driven ATP Synthase Membrane Rotor with a Two-Carboxylate Ion-Coupling Motif
Source: PLoS Biol. 2013 Jun 25;11(6):e1001596. doi: 10.1371/journal.pbio.1001596 (PMC3692424; doi:10.1371/journal.pbio.1001596)
Supplement: Table S1 — Purification of the F. nucleatum F1Fo-ATP synthase. (DOC) [file pbio.1001596.s015.doc]

**Table S1. Purification of the *F. nucleatum* F_1_F_o_**-ATP synthase

| **Step** | **Protein^a^**  **(mg)** | **Activity^b^**  **(Total Units)** | **Specific Activity**  **(U/mg)** | **Purification**  **(fold**) | **Yield**  **(%)** |
| --- | --- | --- | --- | --- | --- |
| **Membrane vesicles** | 142.6 | 21.0 | 0.1 | 1 | 100 |
| **2% DDM solubilizate** | 59.8 | 32.4 | 0.5 | 3.7 | 152 |
| **Anion exchange** | 3.8 | 8.4 | 2.2 | 15.0 | 38 |
| **Size exclusion** | 0.2 | 1.3 | 5.7 | 38.4 | 6 |

^(a)^ The starting material was around 8 g (wet weight) of cells; results are representative of three biological replicates.

^(b)^ The ATPase activity was determined using the ATP-regenerating assay; 1 Unit corresponds to 1 µmol of ATP hydrolysed/minute at 37°C.
