# Supplementary material for: A New Type of Na+-Driven ATP Synthase Membrane Rotor with a Two-Carboxylate Ion-Coupling Motif
Source: PLoS Biol. 2013 Jun 25;11(6):e1001596. doi: 10.1371/journal.pbio.1001596 (PMC3692424; doi:10.1371/journal.pbio.1001596)
Supplement: Table S2 — Collection of X-ray diffracion data and refinement statistics. (DOC) [file pbio.1001596.s016.doc]

Table S2. Collection of X-ray diffracion data and refinement statistics

|  | c_11_, pH 5.3 | | c_11_, pH 8.7 |
| --- | --- | --- | --- |
| **Data collection** |  | |  |
| Beamline | X10SA (PXII), SLS | | ID23.2, ESRF |
| Space group | P2_1_ | | P2_1_ |
| Cell dimensions   |  | |  |
| *a*, *b*, *c* (Å) | 135.8, 84.0, 151.1 | | 136.5, 84.1, 152.1 |
|     *α*, *β*, *γ * (°) | 90.0, 112.8, 90.0 | | 90.0, 112.9, 90.0 |
| Resolution (Å)^a^ | 20-2.22 (2.36-2.22)^*^ | | 50-2.64 (2.80-2.64)^*^ |
| *R*_meas_^#^ | 13.6 (100.8) | | 24.0 (117.4) |
| *R*_mergd-F_^#^ | 20.2 (96.9) | | 28.0 (112.6) |
| *I* / σ*I* | 8.92 (1.56) | | 7.10 (1.57) |
| Completeness (%) | 99.1 (97.3) | | 99.0 (95.4) |
| Redundancy | 4.54 (4.52) | | 4.21 (3.97) |
|  |  | |  |
| **Refinement** |  | |  |
| Resolution (Å) | 29.75-2.22 | | 47.37-2.64 |
| No. reflections | 158181 | | 92184 |
| *^#^R*_work_ / ^#^*R*_free_ | 19.93 / 23.08 | | 18.46 / 23.64 |
| No. atoms | 14836 | | 14537 |
| Protein | 13640 | | 13639 |
| Ligand | 853 | | 759 |
| Water | 321 | | 117 |
| *B*-factors | 40.35 | | 39.87 |
| Protein | 36.74 | | 36.21 |
| Ligand | 96.20 | | 106.34 |
| Water | 46.09 | | 35.84 |
| R.m.s. deviations |  | |  |
| Bond lengths (Å) | 0.004 | | 0.007 |
| Bond angles (°) | 0.854 | | 1.038 |
| Ligands^+^ | 22 DMU, 3 LMT, 2 TAM, 22 Na^+^ | | 23 DMU, 22 Na^+^ |
| ^*^Values in parentheses are for highest-resolution shell. Inclusion of data to 2.22 Å resolution, despite the high R_meas_, R_mergd-F_ and *I*/σ*I* values, resulted in noticeable improvements in the electron density.  ^+^DMU: β-decyl-maltoside, LMT: β-dodecyl-maltoside, TAM: tris(hydroxymethyl)aminomethane.  ^#^Defined by the following equations (Blow, D. Outline of crystallography for biologists. Oxford University Press, New York (2002)): | | | |
| 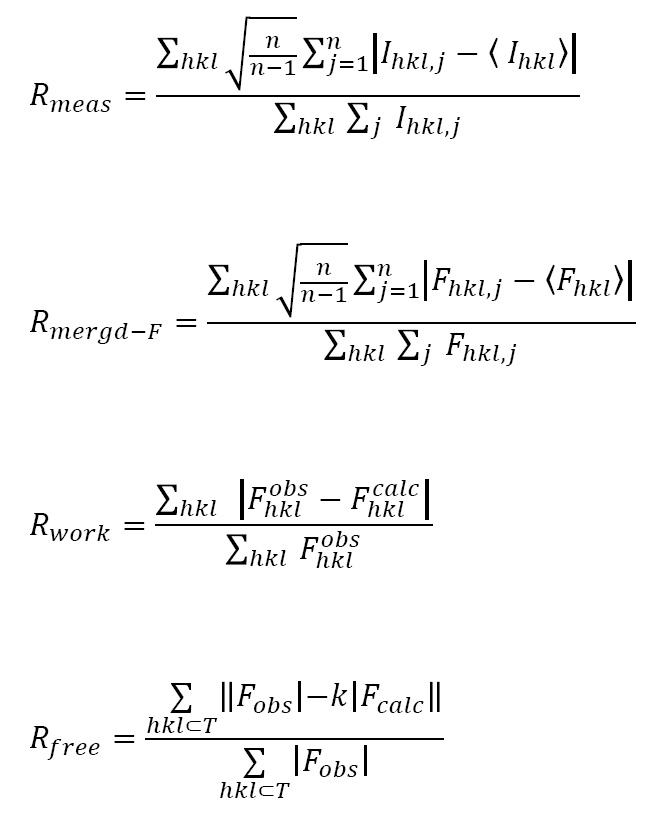 | | 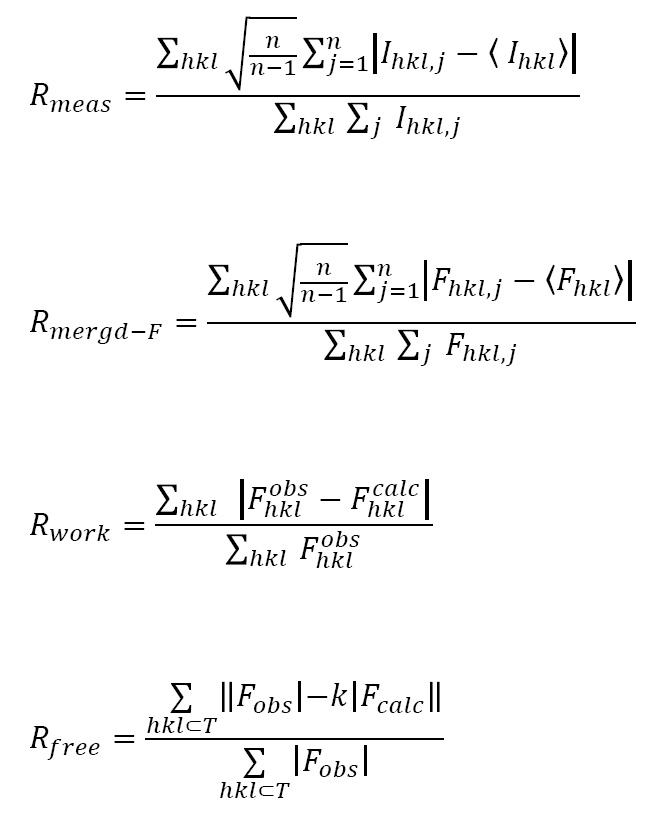 | |
