# Supplementary material for: A New Type of Na+-Driven ATP Synthase Membrane Rotor with a Two-Carboxylate Ion-Coupling Motif
Source: PLoS Biol. 2013 Jun 25;11(6):e1001596. doi: 10.1371/journal.pbio.1001596 (PMC3692424; doi:10.1371/journal.pbio.1001596)
Supplement: Text S1 — Supporting materials and methods, and references. (DOC) [file pbio.1001596.s017.doc]

**Text S1**

**Supplementary Information**

**A new type of Na^+^-driven ATP synthase membrane rotor
with a two-carboxylate ion-coupling motif**

Sarah Schulz^1^, Marina Iglesias-Cans^2^, Alexander Krah^3^,
Özkan Yildiz^1^, Vanessa Leone^3^, Doreen Matthies^1^,
Gregory M. Cook^2^, José D. Faraldo-Gómez^3,4 # *^, Thomas Meier^1,4 # *^

^1^Department of Structural Biology,

Max Planck Institute of Biophysics,

Max-von-Laue-Strasse 3, 60438 Frankfurt am Main, Germany

^2^Department of Microbiology and Immunology,

Otago School of Medical Sciences, University of Otago,

720 Cumberland Street, 9054 Dunedin, New Zealand

^3^Theoretical Molecular Biophysics Group,

Max Planck Institute of Biophysics,

Max-von-Laue-Strasse 3, 60438 Frankfurt am Main, Germany

^4^Cluster of Excellence “Macromolecular Complexes”,

Goethe University of Frankfurt,

Max-von-Laue-Strasse 15, 60438 Frankfurt am Main, Germany

^#^These authors contributed equally

^*^To whom correspondence should be addressed:

José D. Faraldo-Gómez, jose.faraldo@biophys.mpg.de

Thomas Meier, thomas.meier@biophys.mpg.de

**Materials and Methods**

**Molecular modeling and classical simulations**

A c_11_ ring of *F. nucleatum* was modeled by homology with the c_11_ ring of *Ilyobacter tartaricus* , using CHARMM . The resulting c_11_ model was embedded in a hydrated phospholipid membrane (**Fig. S4, S15**) as described previously . Several simulation systems were prepared reflecting different occupancies and conformational states of the ion-binding sites. The total number of atoms in each system is ~100,000. Conventional and free-energy molecular dynamics simulations of these models were carried out using NAMD 2.7 and the CHARMM27/CMAP energy function . All simulations were carried out at constant pressure (1 atm) and temperature (298 K), using a Nose-Hoover/Langevin thermo-barostat. The surface area of the membrane was kept constant at ~69 Å^2^ per lipid, and periodic boundary conditions were used. Electrostatic interactions were computed with the Particle-Mesh-Ewald algorithm, with a real-space cut-off of 12 Å. The same cut-off was used for the van der Waals interactions. The Free-Energy Perturbation method was employed in all ion-selectivity and pK_a_ calculations, as implemented in NAMD 2.7. As is common, we followed a step-wise protocol, using a coupling parameter λ reflecting the alchemical transformation between states. In the ion-selectivity calculations, 32 intermediate steps, or windows were employed, while in the pK_a_ calculations, we used 31. Each window included an initial equilibration of 200 ps, followed by a 1 ns run for data collection. All calculations were carried out in the forward and backward directions. Calculations of the pK_a_ also included a correction on account of the membrane polarizability, computed via Poisson theory, using CHARMM . To relate the computed free energies to values of the ion selectivity relative to a reference system, we followed a thermodynamic framework described previously .

### Quantum mechanical calculations

To determine which of two carboxylate side-chains in the c-ring binding site is most likely to host a H^+^ in the presence of Na^+^, we carried out *ab initio* geometry optimizations and energy calculations for two alternative configurations, shown in Figs. 1A and 1B, as well as for the transition state between them. All calculations were carried out on reduced models of the binding site, depicted in Fig. 2A, using Gaussian09 (Frisch M.J. et al., Gaussian Inc., Wallingford CT, 2009), at the HF/6-31G* level of theory. During the geometry optimizations, only the capping methyl groups and the Cα-atoms were fixed in space. The characteristics of each optimized stationary point were assessed by calculating the corresponding Hessian matrix.

### Culture conditions

*Fusobacterium nucleatum*, subsp. *nucleatum* ATCC25586 was grown anaerobically overnight at 37°C in Columbia broth (Difco) supplemented with 0.5 g/L cysteine-HCl as a reducing agent and resazurin (0.5 mg/mL) as an oxygen indicator (pH 7.5). Cells were routinely grown in either anaerobic gassed (N_2_/H_2_/CO_2_, 90:5:5) Hungate tubes or in 1 L Schott bottles with oxygen-impermeable butyl rubber stoppers. Schott bottle incubations were carried out in an anaerobic chamber (gas composition N_2_/H_2_/CO_2_, 85:10:5) and the initial inoculum was 10% of the culture volume. All inhibitors were dissolved in 0.1 ml of ethanol (or appropriate control of ethanol). Inhibitors or ethanol were added to 10 ml cultures in Hungate tubes in mid exponential phase (Optical density at 600 nm of around 0.4).

### Preparation of inverted membrane vesicles

All steps were carried out at 4°C. Typically, 8 to 10 g of mid-exponential phase cells were washed in buffer (50 mM 3-(*N*-morpholino)propanesulfonic acid (MOPS), 2 mM MgCl_2_, pH 7.5) and resuspended in 20 ml of membrane buffer (50 mM MOPS, 2 mM MgCl_2_, 1 mM dithiothreitol (DTT), Roche protease inhibitor cocktail and 2 mg DNAse, pH 7.5). Cells were disrupted by three passages through a pre-cooled French pressure cell (20,000 p.s.i.). Unbroken cell material was removed via centrifugation at 8’000 × g for 10 min, and the membranes were collected by ultracentrifugation at 150’000 × g for 45 min. Membranes were washed twice in membrane buffer and resuspended in membrane buffer to a final concentration of 20-30 mg/ml.

### Solubilization and purification of the F_1_F_o_-ATPase from *F. nucleatum*

Inverted membrane vesicles were resuspended in solubilization buffer (50 mM MOPS, 2 mM MgCl_2_, 1 mM DTT, 2% β-dodecyl-maltoside (DDM), 1% glycerol, Roche protease inhibitor cocktail, pH 7.5) to a final concentration of 5 mg of protein/ml and incubated at 4°C for 1h with constant stirring. The insoluble material was removed by ultracentrifugation at 150’000 × g for 45 min and the solubilized supernatant was filtered (0.45 µm) and loaded onto a 5 ml anion exchange column (HiTrap^TM^ Q HP, GE Healthcare) that had been equilibrated in buffer A (20 mM tris(hydroxymethyl)aminomethane (Tris)-HCl, 1 mM MgCl_2_, 10% glycerol, 0.05% DDM, pH 8.0). Bound proteins were eluted by a linear gradient of NaCl (from 0 to 1 M with a flow rate of 0.5 ml/min) in the same buffer and 1 ml fractions were collected. The *F. nucleatum* F_1_F_o_-ATP synthase-containing fractions (at around 500 mM NaCl) determined by ATPase activity, were pooled and concentrated using Amicon centrifugal filter units (NMWL, 100’000). The concentrated sample (0.5 ml) was applied to a Superose 6 10/300 (GE Healthcare) equilibrated in size exclusion buffer (20 mM Tris-HCl, 1 mM MgCl_2_, 100 mM NaCl, 0.05% DDM, pH 8.0) and the ATP synthase-containing fractions were determined by ATPase activity. For *F. nucleatum* F_1_F_o_-ATP synthase characterization purposes, the ATPase active fractions pooled from the anion exchange purification were applied to a PD-10 desalting column and resuspended in buffer A free of NaCl (50-100 µM). Proteins were analyzed by 12.5% sodium dodecylsulfate-polyacrylamide gel electrophoresis (SDS-PAGE) using the Lämmli buffer system . Protein bands were visualized by silver staining or Coomassie staining . The identification of the F_1_F_o_-ATP synthase subunits was further confirmed by matrix-assisted laser desorption/ionization tandem time-of-flight mass spectrometry (MALDI TOF/TOF MS) on a 4’800 MALDI TOF/TOF analyzer (AB Sciex, MA, USA) as previously described . Protein concentrations were quantified by the Bradford method following manufacturer instructions (Biorad, Albany, New Zealand) and using bovine serum albumin as the standard.

### ATP hydrolysis and ATP synthesis activity measurements

ATP hydrolysis activity from inverted membranes and purified protein was measured using the spectrophotometric ATP-regenerating assay at 37°C, as described before . The reaction was initiated by the addition of 2.5 mM Na_2_-ATP, and the rate of NADH oxidation was followed continuously at 340 nm with a Cary 50 (Varian Inc.) spectrophotometer. The pH profile of the ATP synthase was characterized in inverted membranes in a three buffer mix composed of 50 mM 2-(*N*-morpholino)ethanesulfonic acid (MES)-MOPS-Tris buffer. When sodium-free assays were required, Tris-ATP was used in place of Na_2_-ATP. When the ATP-regenerating assay could not be used, ATP hydrolysis was measured via the release of P_i_ . The assay solution contained 50 mM MOPS (pH 7.5), 2 mM MgCl_2_, and 5 mM Na_2_-ATP. The amount of non specific P_i_ released during the assay was corrected for and one unit of ATPase activity was defined as the amount of enzyme that liberated one µmol of P_i_ or ADP per min at 37°C.

ATP-driven proton translocation was determined at 37°C by the quenching of acridine orange (AO), as described previously with some modifications; the 2 ml reaction mix contained 5 mM potassium phosphate buffer (pH 6), 5 mM MgCl_2_, 5 µM AO, 100 mM KCl, and membrane vesicles containing 300 mg of protein. The reaction was initiated with sodium-ATP (1.25 mM final concentration) and fluorescence was measured with excitation and emission wavelengths of 492 and 528 nm, respectively (slit width 5 nm). The reaction was reversed by the addition of 30 µM CCCP.

ATP synthesis in inverted membrane vesicles was determined via the standard luciferin-luciferase system, monitoring the light emitted with a chemiluminometer (FB 12 luminometer; Berthold) at 37°C, as described before . The ATP synthesis reactions were carried out at 37°C in a 400-μl volume containing 10 μl of inverted membrane vesicles (K^+^_in_ = 5 mM), 10 mM Tricine-KOH (pH 8.0), 2 mM MgCl_2_, 5 mM KH_2_PO_4_, 2.5 mM ADP, and 200 mM KCl. The synthesis reaction was initiated via the addition of 2 μM valinomycin to induce a potassium diffusion potential of approximately 100 mV as calculated using the Nernst equation: 61 × log_10_ ([K^+^]_out_/[K^+^]_in_). To load inverted membrane vesicles with Na^+^, vesicles were incubated in 50 mM MOPS (pH 7.5) buffer containing 100 mM NaCl overnight at 4°C. To create a chemical gradient of sodium ions of 100 mV (61 × log_10_ ([Na^+^]_out_/[Na^+^]_in_), Na^+^(100 mM)-loaded vesicles were diluted 40-fold into 10 mM Tricine-KOH (pH 8.0), 2 mM MgCl_2_, 5 mM KH_2_PO_4_, and 2.5 mM ADP. To additionally impose a Δψ (100 mV) in the presence of a chemical gradient of sodium ions, 200 mM KCl and valinomycin were included in the dilution buffer. Samples (25 μl) were withdrawn every 5 to 10 s, and the reaction was stopped by diluting the sample with 400 μl of stop mix containing 50 mM MOPS supplemented with 1% TCA and 2 mM EDTA. The luciferase reaction was initiated by adding 40 μl of luciferin-luciferase mix to 40 μl of sample.

**Production and purification of the *F. nucleatum* c-ring**

The c-ring from *F. nucleatum* was produced in a heterologous expression system using the ATP synthase lacking *E. coli* strain DK8 (Δ*atp*) . Plasmid pITtr5His containing the whole *atp* operon from *Ilyobacter tartaricus* with a His_6_-tag at the N-terminus of the β-subunit of the enzyme was modified by replacing the *atpE* gene from *I. tartaricus* with the codon-optimized *atpE* gene from *F. nucleatum.* Cloning was performed using primer containing restriction sites for *Sac*I (forward primer: 5`-CTTTAAGAAGGAGATGAGCTCATGGATCT-3`) and *Cla*I (reverse primer: 5`-CGATAAGGCTTCCTCCATCGATTTAGCCC-3`) for the insertion of the *atpE* gene into the plasmid to create a hybrid ATP synthase. *E. coli* cells were grown in 2 L Terrific Broth (TB) medium at 37°C under continuous shaking at 120 rpm in 5 L Erlenmeyer flasks with baffles until mid-logarithmic phase (OD_600_= 0.6). Expression of the ATP synthase was induced by the addition of 0.5 mM isopropyl β-*D*-1-thiogalactopyranoside. After 4 h of continuous shaking at 37°C the cells were harvested by centrifugation (5’000 g, 30 min, 4°C) and resuspended in 10 ml of 50 mM potassium phosphate buffer (KPB, pH 8.0) per liter culture. Cells were broken using a microfludizer (Microfluidics Corporation, Massachusetts, USA) at 4°C and 1200 bar. Unbroken cells were removed by centrifugation (30’000 g, 30 min, 4°C) and membranes were isolated from the supernatant by ultracentrifugation (200’000 g, 1 h, 4°C). The hybrid ATP synthase was solubilized with 1% (w/v) β-dodecyl-maltoside (DDM, Glycon, Luckenwalde, Germany) at 4°C for 1 h and purified via affinity chromatography using Ni-NTA sepharose (GE Healthcare, Solingen, Germany). The column was equilibrated with 10 column volumes (CV) of buffer (50 mM KPB pH 7.0, 300 mM NaCl, 10% glycerol, 2 mM MgCl_2_, 0.05% DDM, 5 mM imidazole) and washed with a stepwise increase of the imidazole concentration. The hybrid ATP synthase was eluted with 400 mM imdazol in 3 CV. Finally, the purified hybrid ATP synthase was either directly used or stored over night at 4°C and then used for the subsequent purification of the c-ring. The isolation of the c-ring from *F. nucleatum* was carried out as reported previously . After dialysis against 20 mM Tris/HCl pH 8.0, the c-ring was further separated from remaining contaminants by incubating the sample with 1.5% (w/v) Foscholine-12 (Anatrace, Santa Clara, CA, USA) at 60°C for 12 min followed by a sucrose density step gradient using 5-35% sucrose in 20 mM Tris/HCl pH 8.0, 100 mM NaCl, running at 200’000 g for 20 h at 10°C. The c-ring-containing fractions were pooled and directly loaded on a hydroxyapatite column (Meier et al., J. Bacteriol., 2006). The c-ring was eluted with 1 M KPB pH 7.0 containing 0.5% β-decyl-maltoside (DM, Glycon, Luckenwalde, Germany), desalted in 20 mM Tris/HCl pH 8.0, 0.5% (w/v) DM and concentrated using Amicon centrifugation tubes (Merck Millipore, Darmstadt, Germany) with a molecular weight cut-off of 100’000 to a final concentration of 2 mg/ml (bicinchoninic acid protein assay reagent, Thermo Scientific Pierce, St. Leon Rot).

**NCD-4 labeling experiments**

A 60 μl-sample of 0.45 mg/ml *F. nucleatum* c-ring in 20 mM Tris/HCl pH 8.0 and 1.5% (w/v) octyl-glycoside (OG, Glycon, Luckenwalde, Germany) was adjusted to pH 5.7 by the addition of 2-(*N*-morpholino)ethanesulfonic acid (MES/HCl) pH 5.0. Subsequently, the reaction was started with 100 μM NCD-4 (Invitrogen, Darmstadt, Germany) solved in 10% (w/v) DDM. The continuous increase of fluorescence (λ_ex_ = 342 nm, λ_em_ = 452 nm) was monitored with an F-4500 Hitachi Fluorescence Spectrophotometer at 35°C. The reaction was stopped by the addition of 15 mM NaCl (final concentration) to the sample using a 1 M NaCl stock solution.

**Crystallization of the *F. nucleatum* c-ring**

Crystallization of the isolated c-ring was performed by vapor diffusion in hanging drops at 18°C. The sample containing 2 mg/ml of c-ring and 2% (w/v) β-decyl-maltoside (Glycon, Luckenwalde, Germany) was mixed with either crystallization buffer 1 (0.1 M NaAc pH 4.5, 28% (v/v) polyethylene glycol (PEG) 300), or buffer 2 (0.1 M CHES pH 9.6 (NaOH), 27.5 % (v/v) PEG 300, 5 mM MgCl_2_). Crystals grew within one week to a size of ~100×40×40 μm^3^. After three (buffer 1) or four months (buffer 2) of incubation at 18°C, crystals were fished using a nylon loop (Hampton Research Inc. Aliso Viejo, CA, USA) and flash-frozen in liquid nitrogen.

**Data collection, structure determination and refinement**

Data to 2.2 Å (pH 5.3) and 2.64 Å (pH 8.7) were collected at the Max-Planck/Novartis beamline X10SA (PXII) of the Swiss Light Source (SLS, Villigen, Switzerland) and the beamline ID23.2 of the European Synchrotron Radiation Facility (ESRF), Grenoble, France, respectively. The collected data were processed using the XDS software package and the pH 5.3 structure was determined by molecular replacement using PHASER with the c_11_ ring of *I. tartaricus* (PDB code 1YCE ) as a search model. The pH 8.7 structure was determined using the pH 5.3 structure as a search model, with Na^+^ as well as the structural water molecule omitted to prevent model bias, and to explicitly determine the occupancy of the binding sites. In both cases, to avoid model bias we applied density modification and solvent flattening with RESOLVE . Repeating cycles of model building with COOT and refinement with PHENIX and REFMAC followed. The electron density maps after refining were clearly interpretable and correspond to the c_11_ ring of *F. nucleatum,* with 99.7% of the residues within the allowed regions of the Ramachandran plot. Figures were rendered using Pymol . The atomic coordinates and structure factors of the *F. nucleatum* c_11_ ring at pH 5.3 and pH 8.7 were deposited in the Protein Data Bank, under the accession numbers 3ZK1, and 3ZK2, respectively.

**Supplementary References**

1. Meier T, Polzer P, Diederichs K, Welte W, Dimroth P (2005) Structure of the rotor ring of F-type Na^+^-ATPase from *Ilyobacter tartaricus*. Science 308: 659-662.

2. Brooks BR, Brooks CL, Mackerell AD, Nilsson L, Petrella RJ, et al. (2009) CHARMM: The Biomolecular Simulation Program. J Comp Chem 30: 1545-1614.

3. Staritzbichler R, Anselmi C, Forrest LR, Faraldo-Gómez JD (2011) GRIFFIN: A versatile methodology for optimization of protein-lipid interfaces for membrane protein simulations. J Chem Theor Comp 7: 1167-1176.

4. Phillips JC, Braun R, Wang W, Gumbart J, Tajkhorshid E, et al. (2005) Scalable molecular dynamics with NAMD. J Comput Chem 26: 1781-1802.

5. MacKerell AD, Bashford D, Bellott M, Dunbrack RL, Evanseck JD, et al. (1998) All-atom empirical potential for molecular modeling and dynamics studies of proteins. J Phys Chem B 102: 3586-3616.

6. MacKerell AD, Feig M, Brooks CL (2004) Extending the treatment of backbone energetics in protein force fields: limitations of gas-phase quantum mechanics in reproducing protein conformational distributions in molecular dynamics simulations. J Comput Chem 25: 1400-1415.

7. Krah A, Pogoryelov D, Langer JD, Bond PJ, Meier T, et al. (2010) Structural and energetic basis for H^+^ versus Na^+^ binding selectivity in ATP synthase F_o_ rotors. Biochim Biophys Acta 1797: 763-772.

8. Lämmli UK (1970) Cleavage of structural proteins during assembly of head of bacteriophage T4. Nature 227: 680-685.

9. Nesterenko MV, Tilley M, Upton SJ (1994) A simple modification of Blum's silver stain method allows for 30 minute detection of proteins in polyacrylamide gels. J Biochem Bioph Meth 28: 239-242.

10. Fazekas SDS, Webster RG, Datyner A (1963) Two new staining procedures for quantitative estimation of proteins on electrophoretic strips. Biochim Biophys Acta 71: 377-&.

11. McMillan DG, Ferguson SA, Dey D, Schröder K, Aung HL, et al. (2011) A_1_A_o_-ATP synthase of *Methanobrevibacter ruminantium* couples sodium ions for ATP synthesis under physiological conditions. J Biol Chem 286: 39882-39892.

12. Ferguson SA, Keis S, Cook GM (2006) Biochemical and molecular characterization of a Na^+^-translocating F_1_F_o_-ATPase from the thermoalkaliphilic bacterium *Clostridium paradoxum*. J Bacteriol 188: 5045-5054.

13. Kobayashy H, Anraku Y (1972) Membrane-bound adenosine-triphosphatase of *Escherichia coli*. 1. Partial purification and properties. J Biochem 71: 387-399.

14. Keis S, Stocker A, Dimroth P, Cook GM (2006) Inhibition of ATP hydrolysis by thermoalkaliphilic F_1_F_o_-ATP synthase is controlled by the C-terminus of the ε subunit. J Bacteriol 188: 3796-3804.

15. Klionsky DJ, Brusilow WSA, Simoni RD (1984) In vivo evidence for the role of the ε subunit as an inhibitor of the proton-translocating ATPase of *Escherichia coli*. J Bacteriol 160: 1055-1060.

16. Vorburger T, Ebneter JZ, Wiedenmann A, Morger D, Weber G, et al. (2008) Arginine-induced conformational change in the c-ring/a-subunit interface of ATP synthase. FEBS J 275: 2137-2150.

17. Tartoff KD, Hobbs CA (1987) Improved media for growing plasmid and cosmid clones. Bethesda Res Lab Focus 9: 12.

18. Meier T, Matthey U, von Ballmoos C, Vonck J, von Nidda TK, et al. (2003) Evidence for structural integrity in the undecameric c-rings isolated from sodium ATP synthases. J Mol Biol 325: 389-397.

19. Kabsch W (1993) Automatic processing of rotation diffraction data from crystals of initially unknown symmetry and cell constants. J Appl Crystallogr 26: 795-800.

20. McCoy AJ (2007) Solving structures of protein complexes by molecular replacement with Phaser. Acta Crystallogr D 63: 32-41.

21. Terwilliger T (2004) SOLVE and RESOLVE: automated structure solution, density modification, and model building. J Synchrotron Radiat 11: 49-52.

22. Emsley P, Cowtan K (2004) Coot: model-building tools for molecular graphics. Acta Crystallogr D 60: 2126-2132.

23. Zwart PH, Afonine PV, Grosse-Kunstleve RW, Hung LW, Ioerger TR, et al. (2008) Automated structure solution with the PHENIX suite. Methods Mol Biol 426: 419-435.

24. Murshudov GN, Vagin AA, Dodson EJ (1997) Refinement of macromolecular structures by the maximum-likelihood method. Acta Crystallogr D 53: 240-255.

25. DeLano WL (2009) PyMOL molecular viewer: Updates and refinements. Abstr Pap Am Chem S 238.

26. Murata T, Yamato I, Kakinuma Y, Leslie AGW, Walker JE (2005) Structure of the rotor of the V-type Na^+^-ATPase from *Enterococcus hirae*. Science 308: 654-659.
